# Supplementary material for: Heavy isotope labeling and mass spectrometry reveal unexpected remodeling of bacterial cell wall expansion in response to drugs
Source: eLife. 2022 Jun 9;11:e72863. doi: 10.7554/eLife.72863 (PMC9249393; doi:10.7554/eLife.72863)
Supplement: Supplementary file 1. — (a) Muropeptide composition of the peptidoglycan of strains BW25113∆6ldt, M1.5, and BW25113 ‘wild-type’. aData are the mean ± standard deviation from independent experiments (see note c). bβ-Lactam added to the growth medium. AZT, aztreonam at 12 µg/mL; MEC, mecillinam at 2.5 µg/mL; AMP, ampicillin at 16 µg/mL. cThe number of independent peptidoglycan analyses is indicated in parenthesis. Abbreviations: ND, not detected; Tri, tripeptide monomer; Tetra, tetrapeptide monomer; Penta, pentapeptide monomer; Tri→Tri, Tri→Tetra, and Tri→Penta, 3→3 cross-linked dimers; Tetra→Tri and Tetra→Tetra, 4→3 cross-linked dimers. The inter-peptide cross-link direction is indicated using the donor→acceptor conventional notation. (b) Generation time for growth of BW25113 derivatives in M1 minimal medium aβ-lactam added to the growth medium. AZT, aztreonam at 12 µg/mL; MEC, mecillinam at 2.5 µg/mL; AMP, ampicillin at 16 µg/mL. bFor growth in the presence of aztreonam and mecillinam, the values were deduced from the variation in OD600 during 90 min. (c) Susceptibility of Escherichia coli strains to β-lactams. Data are the medians from six experiments. [file elife-72863-supp1.docx]

**SUPPLEMENTARY TABLES**

| **Supplementary file 1a; Table S1. Muropeptide composition of the peptidoglycan of strains BW25113**$\boldsymbol{\Delta}$**6*ldt*, M1.5, and BW25113 “wild type”** | | | | | | | | | | |  |  |
| --- | --- | --- | --- | --- | --- | --- | --- | --- | --- | --- | --- | --- |
| **Strain** | | **Muropeptide (%) ^a^** | | | | | | | | | | |
| **β-lactam ^b^** | | **Tri** | | **Tetra** | **Penta** | **Tri**$\boldsymbol{\to}$**Tri** | **Tri**$\boldsymbol{\to}$**Tetra** | **Tri**$\boldsymbol{\to}$**Penta** | **Tetra**$\boldsymbol{\to}$**Tri** | **Tetra**$\boldsymbol{\to}$**Tetra** | |  |
| BW25113Δ6*ldt* | | | | |  |  |  |  |  |  | |  |
|  | None (9) ^c^ | ND | | 74 $\pm$ 2 | ND | ND | ND | ND | ND | 26 $\pm$ 2 | |  |
|  | AZT (5) ^c^ | ND | | 77 $\pm$ 1 | ND | ND | ND | ND | ND | 23 $\pm$ 1 | |  |
|  | MEL (5) ^c^ | ND | | 70 $\pm$ 2 | ND | ND | ND | ND | ND | 30 $\pm$ 2 | |  |
| M1.5 | |  |  |  |  |  |  |  |  |  | |  |
|  | None (12) ^c^ | 31 $\pm$ 3 | | 36 $\pm$ 4 | ND | 7 $\pm$ 1 | 8 $\pm$ 1 | ND | 7 $\pm$ 1 | 12 $\pm$ 1 | |  |
|  | AMP (10) ^c^ | 29 $\pm$ 2 | | 21 $\pm$ 3 | 6 $\pm$ 2 | 13 $\pm$ 2 | 24 $\pm$ 1 | 6 $\pm$ 0.4 | 1 $\pm$ 0.3 | ND | |  |
| BW25113 "Wild type" | | | | |  |  |  |  |  |  | |  |
|  | None (5) ^c^ | 24 ± 4 | | 48 ± 6 | ND | ND | 2 ± 1 | ND | 2 ± 0.2 | 23 ± 3 | |  |
|  | AZT (5) ^c^ | 14 ± 5 | | 61 ± 3 | ND | ND | 3 ± 0.3 | ND | 3 ± 0.3 | 19 ± 3 | |  |
|  | MEL (5) ^c^ | 28 ± 8 | | 46 ± 9 | ND | ND | 3 ± 0.3 | ND | 3 ± 1 | 20 ± 1 | |  |

^a^ Data are the mean ± standard deviation from independent experiments (see note c).

^b^ β-lactam added to the growth medium. AZT, aztreonam at 12 µg/mL; MEC, mecillinam at 2.5 µg/mL; AMP, ampicillin at 16 µg/mL.

^c^ The number of independent peptidoglycan analyses is indicated in parenthesis.

**Abbreviations:** ND, not detected; Tri, tripeptide monomer; Tetra, tetrapeptide monomer; Penta, pentapeptide monomer; Tri→Tri, Tri→Tetra, and Tri→Penta, 3→3 cross-linked dimers; Tetra→Tri and Tetra→Tetra, 4→3 cross-linked dimers. The inter-peptide cross-link direction is indicated using the donor→acceptor conventional notation.

| **Supplementary file 1b; Table S2. Generation time for growth of BW25113 derivatives in M1 minimal medium** | | | |
| --- | --- | --- | --- |
| **Strain** | | **Generation time (min)** | |
|  | **β-lactam ^a^** | **Experiment 1** | **Experiment 2** |
| M1.5 | |  |  |
|  | None | 90 ± 2 | 85 ± 3 |
|  | AMP | 163 ± 9 | 165 ± 13 |
| BW25113Δ6*ldt* | |  |  |
|  | None | 67 ± 1 | 66 ± 1 |
|  | AZT **^b^** | 64 ± 1 | 63 ± 1 |
|  | MEL **^b^** | 63 ± 1 | 65 ± 1 |
| BW25113 "W ild type" | |  |  |
|  | None | 63 ± 8 | 64 ± 8 |
|  | AZT **^b^** | 74 ± 5 | 63 ± 8 |
|  | MEL **^b^** | 55 ± 2 | 58 ± 6 |

^a^ β-lactam added to the growth medium. AZT, aztreonam at 12 µg/mL; MEC, mecillinam at 2.5 µg/mL; AMP, ampicillin at 16 µg/mL.

^b^ For growth in the presence of aztreonam and mecillinam, the values were deduced from the variation in OD_600_ during 90 min.

| **Supplementary file 1c; Table S3. Susceptibility of *E. coli* strains to β-lactams** | | |
| --- | --- | --- |
|  | MIC (µg/mL) | |
| Strain | Aztreonam | Mecillinam |
| BW25113 | 0.0625 | 0.125 |
| BW25113Δ6*ldt* | 0.0625 | 0.125 |
| Data are the medians from six experiments. | | |
