# Supplementary material for: Heavy isotope labeling and mass spectrometry reveal unexpected remodeling of bacterial cell wall expansion in response to drugs
Source: eLife. 2022 Jun 9;11:e72863. doi: 10.7554/eLife.72863 (PMC9249393; doi:10.7554/eLife.72863)
Supplement: Supplementary file 2. [file elife-72863-supp2.pdf]

Supplementary Data

Tri - all heavy . . . 2
Tri - h1 . . . 4
Tri - h2 . . . 6
Tri - h3 . . . 8
Tetra - all heavy . . . 10
Tetra - h1 . . . 12
Tetra - h2 . . . 14
Tetra - h3 . . . 16
Tetra - hAla . . . 18
Tetra - h1h2 . . . 20
Tetra - h3Ala . . . 22
Additional monomer hybrids . . . 24
Tri-Tri - labeled . . . 25
Tri-Tri - unlabeled . . . 27
Tri-Tri - Donor all light . . . 29
Tetra-Tri - labeled . . . 31
Tetra-Tri - unlabeled . . . 33
Tetra-Tri - Donor all light . . . 35
Tri-Tetra - labeled . . . 37
Tri-Tetra - unlabeled . . . 39
Tri-Tetra - Donor all light . . . 41
Tetra-Tetra - labeled . . . 43
Tetra-Tetra - unlabeled . . . 45
Tetra-Tetra - Donor all light . . . 47

Monomers

Supplementary Data 1.1: Tandem mass spectrometry analysis of the labeled disaccharide-tripeptide. The observed m/z_{obs} and calculated m/z_{cal} values of the parental ion, $[M+H]^+$, as determined in the absence of fragmentation (MS1), are indicated. The m/z_{calc} value was used to select the ion for fragmentation. The difference between the m/z_{obs} and m/z_{calc} values is indicated in ppm. The GlcNAc residue is indicated in two moieties, glucosamine (GlcN) and the acetyl group (Ac), since these moieties are potentially differentially labeled. For the same reason, the reduced (Red) MurNAc residue is indicated in three moieties, reduced glucosamine (GlcN^{Red}), the acetyl group (Ac), and the D-lactoyl group. Labeled and unlabeled moieties are represented in red and purple, respectively. The peaks corresponding to the list of fragments appearing in the table are highlighted by orange dots in the mass spectrum. In the mass spectrum, peaks differing by the loss of H_20 are connected by a dashed line. The tables only contain the m/z values for the fragments containing H_20.
An interactive report of the MS^2 analysis is available in Supplementary File F1.1 .

Precursor ion (MS1)	m/z_{obs}	m/z_{calc}	ppm
GlcN(-Ac)-GlcN^{Red}(-Ac)-Lac-Ala-Glu-DAP	911.475	911.475	0.3

Product ion	m/z_{obs}	m/z_{calc}	ppm	Intensity (a.u.)	Isotopologue
GlcN^{Red}(-Ac)-Lac-Ala-Glu-DAP	699.371	699.371	0.7	7650	all heavy
GlcN^{Red}(-Ac)-Lac-Ala-Glu	500.257	500.259	2.7	570	all heavy
Lac-Ala-Glu-DAP	485.254	485.253	-2.7	220	all heavy
Ala-Glu-DAP	410.220	410.221	3.7	3226	all heavy
GlcN^{Red}(-Ac)-Lac-Ala	365.201	365.202	2.5	4356	all heavy
Glu-DAP	335.177	335.177	0.3	9504	all heavy
GlcN^{Red}(-Ac)-Lac	290.158	290.158	-1.0	1835	all heavy
GlcN^{Red}(-Ac)	215.127	215.127	0.5	229	all heavy
GlcN(-Ac)	213.110	213.111	3.7	108	all heavy
DAP	200.122	200.121	-4.1	3273	all heavy

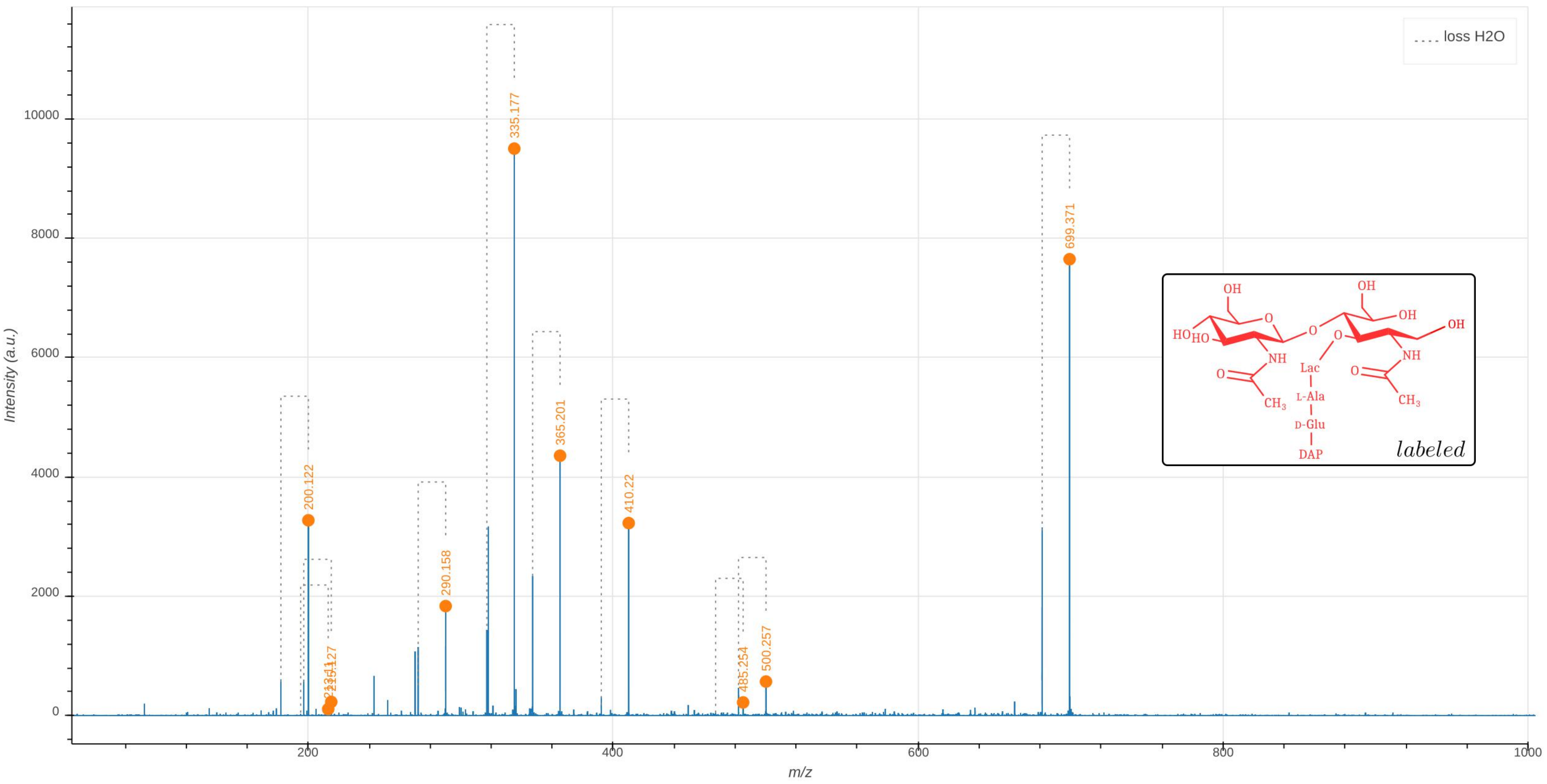

loss H2O
Intensity (a.u.)
m/z
10000
8000
6000
4000
2000
0
200
400
600
800
1000
335.177
699.371
365.201
200.122
410.22
290.158
500.257
485.254
215.127
OH
O
HO
HO
NH
O
CH3
O
OH
OH
OH
O
NH
O
CH3
Lac
L-Ala
D-Glu
DAP
labeled

Supplementary Data 1.2: Tandem mass spectrometry analysis of the h1-type hybrid of the disaccharide-tripeptide. The molecule is fully unlabeled except for one of the two glucosamine moieties present in GlcNAc or MurNAc leading to the presence of two isotopomers. The observed m/z_{obs} and calculated m/z_{calc} values of the parental ion, $[\text{M+H}]^+$, as determined in the absence of fragmentation (MS^1), are indicated. The m/z_{cal} value was used to select the ion for fragmentation. The difference between the m/z_{obs} and m/z_{cal} values is indicated in ppm. The GlcNAc residue is indicated in two moieties, glucosamine (GlcN) and the acetyl group (Ac), since these moieties are potentially differentially labeled. For the same reason, the reduced (Red) MurNAc residue is indicated in three moieties, reduced glucosamine (GlcN^{Red}), the acetyl group (Ac), and the D-lactoyl group. Labeled and unlabeled moieties are represented in red and purple, respectively. The fragmentation led to two series of fragments specific of the isotopomers containing the unlabeled glucosamine moiety in the GlcNAc (h1G) or $\text{MurNAc}^{\text{Red}}$ (h1M) residues, respectively (discriminatory fragments). The fragments specific of h1G and of h1M are highlighted by orange and green dots in the mass spectrum. The other fragments are common to the fragmentation patterns of h1G and of h1M. The corresponding peaks are highlighted by blue dots in the mass spectrum. The mass spectrum indicates the presence of both isotopomers as expected from the recycling and synthesis pathways since UDP-MurNAc exclusively derives from UDP-GlcNAc. In the mass spectrum, peaks differing by the loss of H_20 are connected by a dashed line. The tables only contain the m/z values for the fragments containing H_20.An interactive report of the MS^2 analysis is available in Supplementary File F1.2 .

Precursor Ion (MS^1)	m/z_{obs}	m/z_{calc}	ppm
GlcN(-Ac)-GlcN^{Red}(-Ac)-Lac-Ala-Glu-DAP	878.392	878.396	-4.0
GlcN(-Ac)-GlcN^{Red}(-Ac)-Lac-Ala-Glu-DAP	878.392	878.396	-4.0

Discriminatory product ion	m/z_{obs}	m/z_{calc}	ppm	Intensity (a.u.)	Isotopomer
GlcN^{Red}(-Ac)-Lac-Ala-Glu-DAP	675.318	675.316	-2.3	10563	h1M
GlcN^{Red}(-Ac)-Lac-Ala-Glu-DAP	668.299	668.299	0.6	8694	h1G
GlcN^{Red}(-Ac)-Lac-Ala-Glu	485.221	485.221	0.3	952	h1M
GlcN^{Red}(-Ac)-Lac-Ala-Glu	478.202	478.204	3.4	662	h1G
GlcN^{Red}(-Ac)-Lac-Ala	356.177	356.178	3.6	7236	h1M
GlcN^{Red}(-Ac)-Lac-Ala	349.161	349.161	1.1	6203	h1G
GlcN^{Red}(-Ac)-Lac	285.140	285.141	4.9	3223	h1M
GlcN^{Red}(-Ac)-Lac	278.123	278.124	4.0	3059	h1G
GlcN^{Red}(-Ac)	213.118	213.120	8.2	247	h1M
GlcN(-Ac)	211.106	211.104	-6.8	238	h1G
GlcN^{Red}(-Ac)	206.103	206.103	-0.5	148	h1G
GlcN(-Ac)	204.084	204.087	15.1	419	h1M

Common product ion	m/z_{obs}	m/z_{calc}	ppm	Intensity (a.u.)
GlcN(-Ac)-GlcN^{Red}(-Ac)-Lac-Ala-Glu-DAP	878.397	878.396	-2.1	178
GlcN(-Ac)-GlcN^{Red}(-Ac)-Lac-Ala-Glu-DAP				
GlcN(-Ac)-GlcN^{Red}(-Ac)-Lac-Ala	559.257	559.258	0.8	112
GlcN(-Ac)-GlcN^{Red}(-Ac)-Lac-Ala				
Lac-Ala-Glu-DAP	463.203	463.204	1.8	613
Ala-Glu-DAP	391.182	391.183	2.0	9574
Glu-DAP	320.146	320.146	0.3	26374
Ala-Glu	201.085	201.088	13.5	227
DAP	191.103	191.103	0.6	6654
Lac-Ala	144.068	144.066	-10.4	154

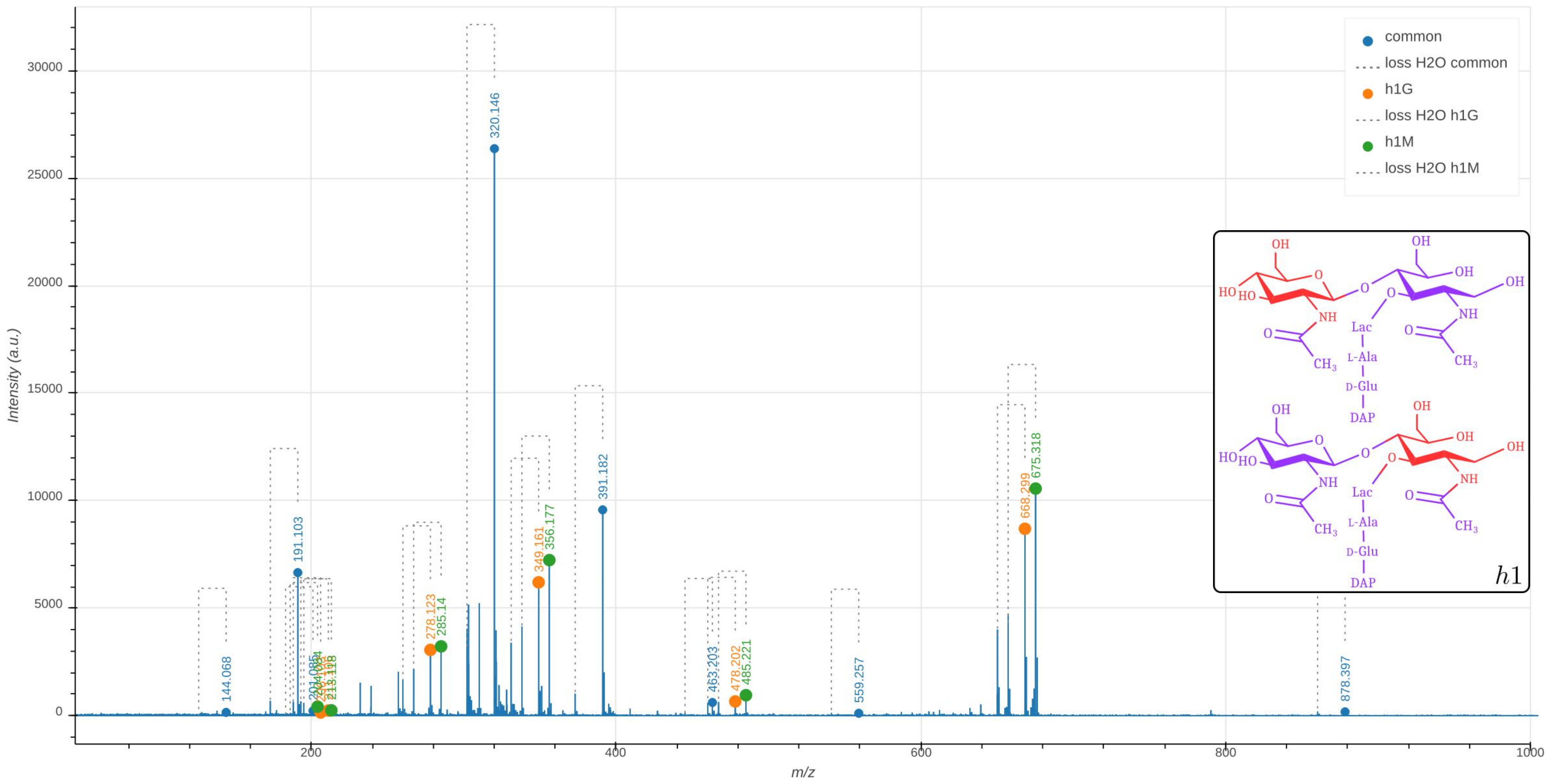
common
loss H2O common
h1G
loss H2O h1G
h1M
loss H2O h1M
Intensity (a.u.)
m/z
320.146
391.182
191.103
144.068
349.161
356.177
278.123
285.14
463.203
478.202
485.221
559.257
668.299
675.318
878.397
h1
Lac
L-Ala
D-Glu
DAP

Supplementary Data 1.3: Tandem mass spectrometry analysis of the h2-type hybrid of the disaccharide-tripeptide. The observed m/z_{obs} and calculated m/z_{cal} values of the parental ion, $[\mathrm{M+H}]^+$, as determined in the absence of fragmentation (MS1), are indicated. The m/z_{calc} value was used to select the ion for fragmentation. The difference between the m/z_{obs} and m/z_{calc} values is indicated in ppm. The GlcNAc residue is indicated in two moieties, glucosamine (GlcN) and the acetyl group (Ac), since these moieties are potentially differentially labeled. For the same reason, the reduced (Red) MurNAc residue is indicated in three moieties, reduced glucosamine ($\mathrm{GlcN^{Red}}$), the acetyl group (Ac), and the D-lactoyl group. Labeled and unlabeled moieties are represented in red and purple, respectively. The peaks corresponding to the list of fragments appearing in the table are highlighted by orange dots in the mass spectrum. In the mass spectrum, peaks differing by the loss of H_20 are connected by a dashed line. The tables only contain the m/z values for the fragments containing H_20.
An interactive report of the MS^2 analysis is available in Supplementary File F1.3 .

Precursor ion (MS1)	m/z_{obs}	m/z_{calc}	ppm
GlcN(-Ac)-$\mathrm{GlcN^{Red}}$(-Ac)-Lac-Ala-Glu-DAP	890.412	890.417	-5.7

Product ion	m/z_{obs}	m/z_{calc}	ppm	Intensity (a.u.)	Isotopologue
$\mathrm{GlcN^{Red}}$(-Ac)-Lac-Ala-Glu-DAP	687.336	687.338	2.2	7465	h2
$\mathrm{GlcN^{Red}}$(-Ac)-Lac-Ala-Glu	488.223	488.225	3.1	404	h2
Lac-Ala-Glu-DAP	482.239	482.242	7.1	296	h2
Ala-Glu-DAP	410.219	410.221	5.5	3431	h2
$\mathrm{GlcN^{Red}}$(-Ac)-Lac-Ala	353.167	353.168	4.7	4771	h2
Glu-DAP	335.178	335.177	-2.4	9561	h2
$\mathrm{GlcN^{Red}}$(-Ac)-Lac	278.123	278.124	2.9	1727	h2
GlcN(-Ac)	204.086	204.087	5.9	190	h2
DAP	200.120	200.121	4.2	2800	h2

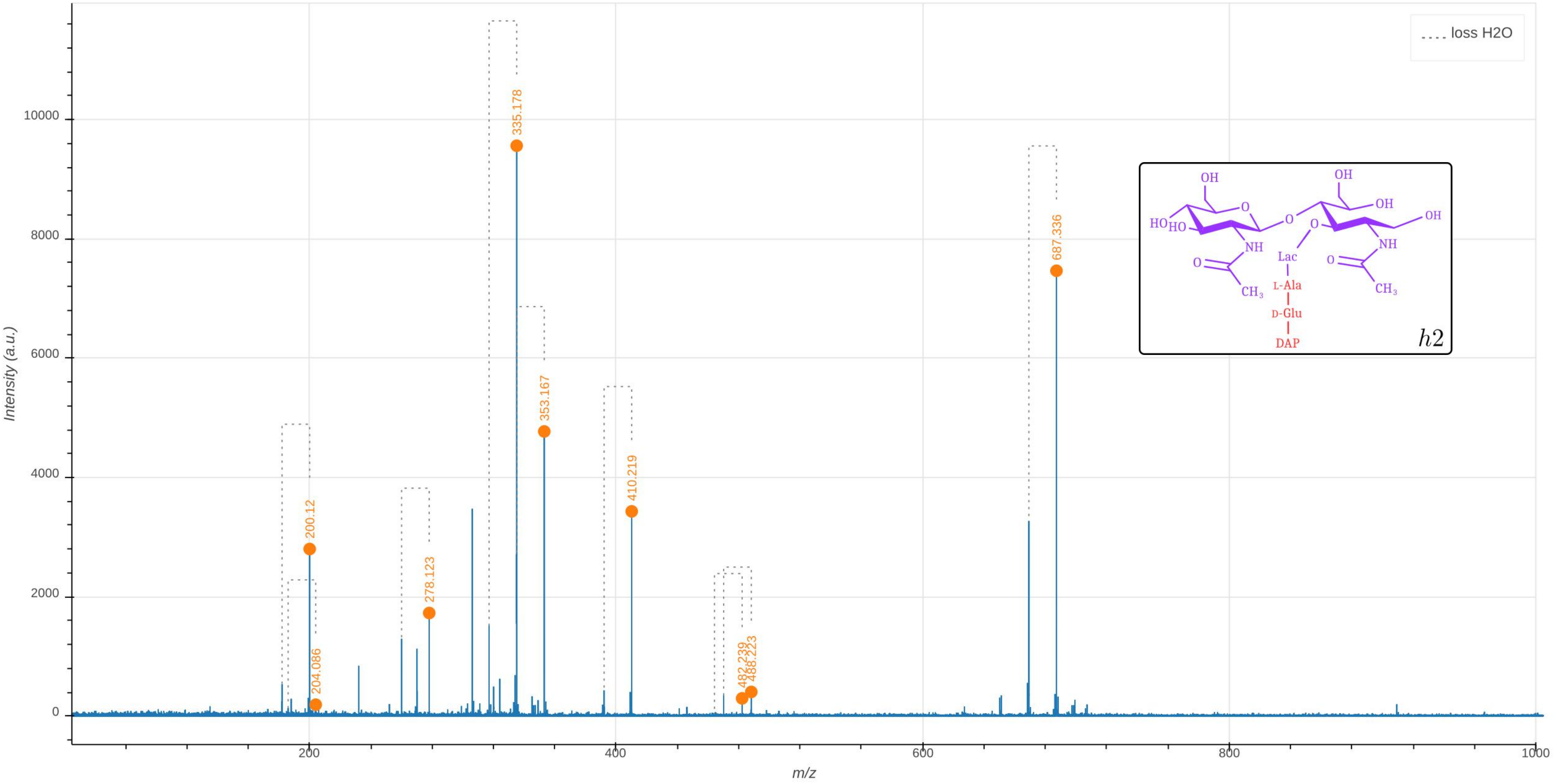

loss H2O
335.178
353.167
410.219
687.336
200.12
204.086
278.123
482.239
488.223
Intensity (a.u.)
m/z
0
2000
4000
6000
8000
10000
200
400
600
800
1000
OH
HO
HO
O
NH
CH3
Lac
L-Ala
D-Glu
DAP
h2

Supplementary Data 1.4: Tandem mass spectrometry analysis of the h3-type hybrid of the disaccharide-tripeptide. The observed m/z_{obs} and calculated m/z_{cal} values of the parental ion, $[\text{M+H}]^+$, as determined in the absence of fragmentation (MS1), are indicated. The m/z_{calc} value was used to select the ion for fragmentation. The difference between the m/z_{obs} and m/z_{calc} values is indicated in ppm. The GlcNAc residue is indicated in two moieties, glucosamine (GlcN) and the acetyl group (Ac), since these moieties are potentially differentially labeled. For the same reason, the reduced (Red) MurNAc residue is indicated in three moieties, reduced glucosamine (GlcN^{Red}), the acetyl group (Ac), and the D-lactoyl group. Labeled and unlabeled moieties are represented in red and purple, respectively. The peaks corresponding to the list of fragments appearing in the table are highlighted by orange dots in the mass spectrum. In the mass spectrum, peaks differing by the loss of H_20 are connected by a dashed line. The tables only contain the m/z values for the fragments containing H_20. The presence of the h3 isotopomer is expected since cells of *E. coli* contain the main PG precursor, UDP-MurNAc-pentapeptide in considerable amounts (about 2% compared to the total amount of dissaccharide peptides in the cell wall). An interactive report of the MS^2 analysis is available in Supplementary File F1.4 .

Precursor ion (MS1)	m/z_{obs}	m/z_{calc}	ppm
GlcN(-Ac)-GlcN^{Red}(-Ac)-Lac-Ala-Glu-DAP	902.447	902.451	-3.8

Product ion	m/z_{obs}	m/z_{calc}	ppm	Intensity (a.u.)	Isotopomer
GlcN^{Red}(-Ac)-Lac-Ala-Glu-DAP	699.372	699.371	-0.5	4005	h3
GlcN^{Red}(-Ac)-Lac-Ala-Glu	500.259	500.259	-0.8	329	h3
Lac-Ala-Glu-DAP	485.251	485.253	3.1	308	h3
Ala-Glu-DAP	410.219	410.221	5.1	1961	h3
GlcN^{Red}(-Ac)-Lac-Ala	365.199	365.202	8.5	2535	h3
Glu-DAP	335.178	335.177	-2.9	5470	h3
GlcN^{Red}(-Ac)-Lac	290.158	290.158	0.2	1047	h3
GlcN(-Ac)	204.088	204.087	-2.6	132	h3
DAP	200.120	200.121	3.8	1331	h3

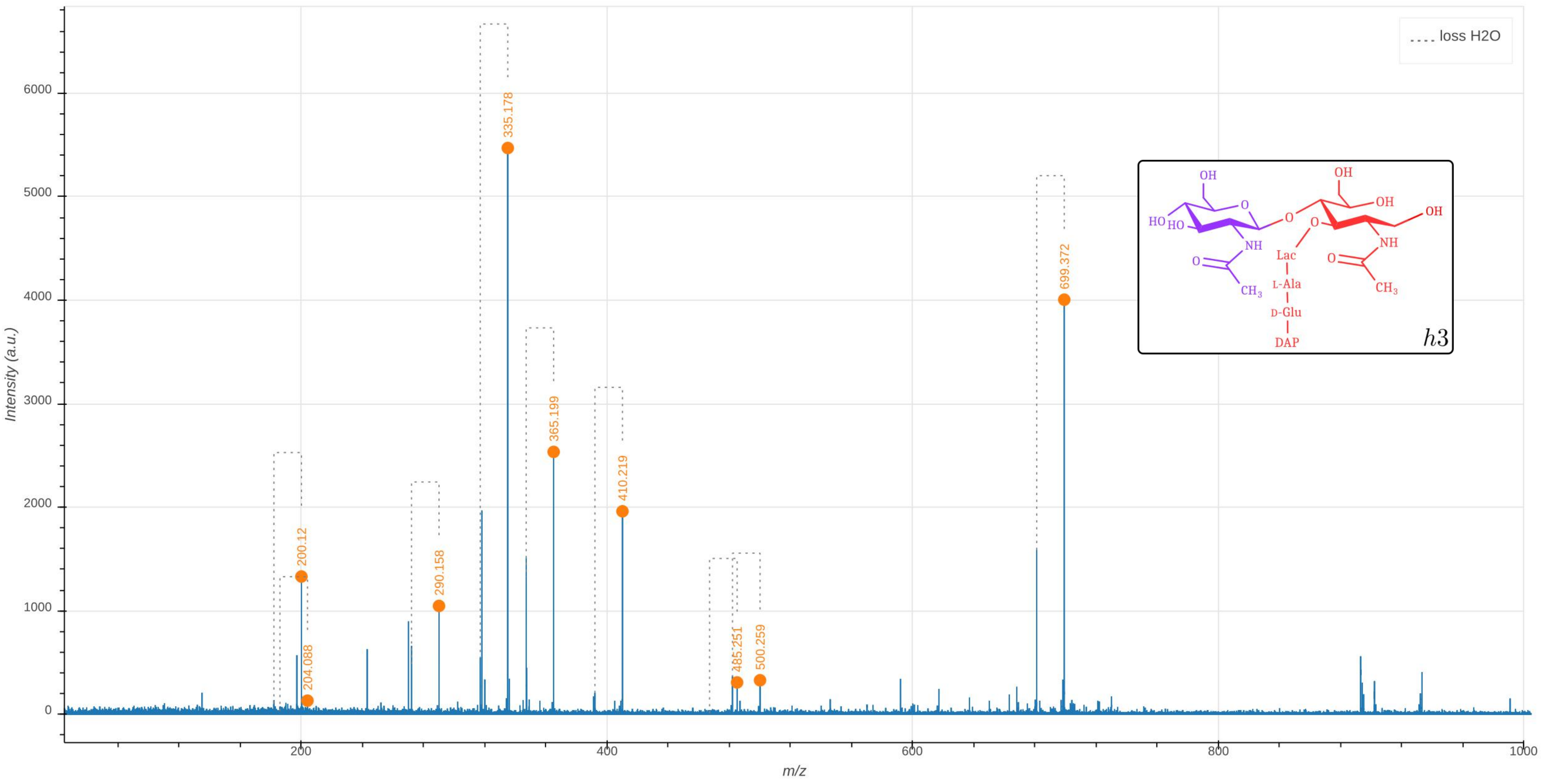
loss H2O
Intensity (a.u.)
m/z
6000
5000
4000
3000
2000
1000
0
200
400
600
800
1000
335.178
699.372
365.199
410.219
200.12
290.158
204.088
485.251
500.259
OH
O
HO
HO
NH
O
CH3
OH
OH
O
O
OH
NH
O
CH3
Lac
L-Ala
D-Glu
DAP
h3

Supplementary Data 2.1: Supplementary Data D2.1 . Tandem mass spectrometry analysis of the labeled disaccharide-tetrapeptide.

The observed m/z_{obs} and calculated m/z_{cal} values of the parental ion, $[M{+}H]^{+}$, as determined in the absence of fragmentation (MS1), are indicated. The m/z_{calc} value was used to select the ion for fragmentation. The difference between the m/z_{obs} and m/z_{calc} values is indicated in ppm. The GlcNAc residue is indicated in two moieties, glucosamine (GlcN) and the acetyl group (Ac), since these moieties are potentially differentially labeled. For the same reason, the reduced (Red) MurNAc residue is indicated in three moieties, reduced glucosamine (GlcN^{Red}), the acetyl group (Ac), and the D-lactoyl group. Labeled and unlabeled moieties are represented in red and purple, respectively. The peaks corresponding to the list of fragments appearing in the table are highlighted by orange dots in the mass spectrum. In the mass spectrum, peaks differing by the loss of H_20 are connected by a dashed line. The tables only contain the m/z values for the fragments containing H_20.

An interactive report of the MS2 analysis is available in Supplementary File F2.1.

Precursor ion (MS1)	m/z_{obs}	m/z_{calc}	ppm
GlcN(-Ac)-GlcN^{Red}(-Ac)-Lac-Ala-Glu-DAP-Ala	986.516	986.519	-3.1

Product ion	m/z_{obs}	m/z_{calc}	ppm	Intensity (a.u.)	Isotopologue
GlcN^{Red}(-Ac)-Lac-Ala-Glu-DAP-Ala	774.423	774.416	-9.3	5487	all heavy
GlcN^{Red}(-Ac)-Lac-Ala-Glu-DAP	681.364	681.361	-5.1	494	all heavy
Lac-Ala-Glu-DAP-Ala	560.296	560.297	0.9	119	all heavy
GlcN^{Red}(-Ac)-Lac-Ala-Glu	500.259	500.259	0.0	194	all heavy
Ala-Glu-DAP-Ala	485.269	485.266	-6.8	1147	all heavy
Glu-DAP-Ala	410.223	410.221	-3.9	3963	all heavy
Ala-Glu-DAP	392.213	392.211	-5.6	1512	all heavy
GlcN^{Red}(-Ac)-Lac-Ala	365.204	365.202	-5.1	2434	all heavy
Glu-DAP	317.169	317.167	-8.5	3861	all heavy
GlcN^{Red}(-Ac)-Lac	290.160	290.158	-7.3	414	all heavy
DAP-Ala	275.167	275.165	-6.2	3667	all heavy
DAP	182.112	182.110	-8.1	611	all heavy

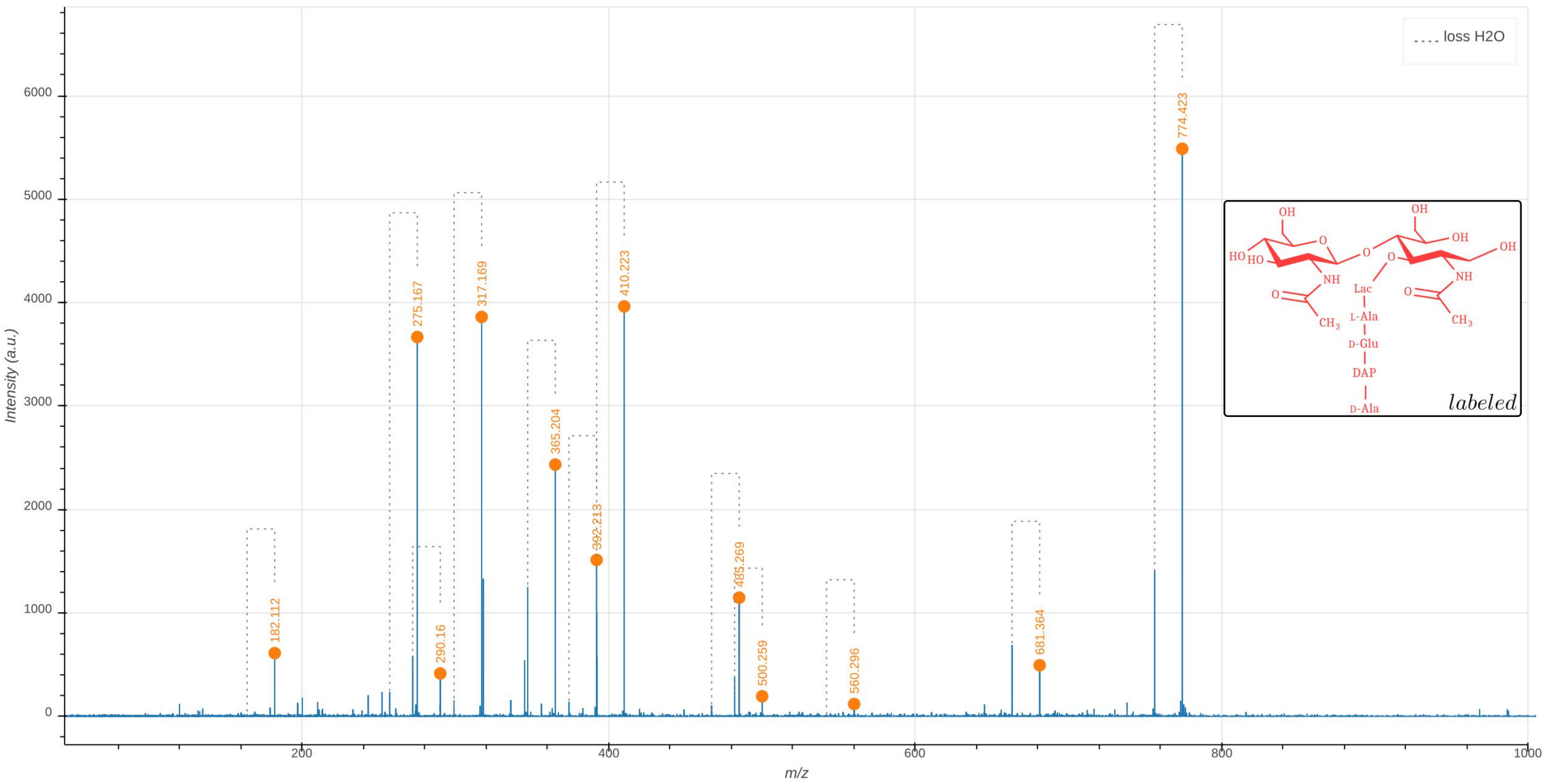

loss H2O
774.423
410.223
317.169
275.167
365.204
392.213
485.269
182.112
290.16
500.259
560.296
681.364
Intensity (a.u.)
m/z
labeled
Lac
L-Ala
D-Glu
DAP
D-Ala

Supplementary Data 2.2: Tandem mass spectrometry analysis of the h1-type hybrid of the disaccharide-tetrapeptide. The molecule is fully unlabeled except for one of the two glucosamine moieties present in GlcNAc or MurNAc leading to the presence of two isotopomers. The observed m/z_{obs} and calculated m/z_{calc} values of the parental ion, $[\mathrm{M+H}]^+$, as determined in the absence of fragmentation (MS^1), are indicated. The m/z_{cal} value was used to select the ion for fragmentation. The difference between the m/z_{obs} and m/z_{cal} values is indicated in ppm. The GlcNAc residue is indicated in two moieties, glucosamine (GlcN) and the acetyl group (Ac), since these moieties are potentially differentially labeled. For the same reason, the reduced (Red) MurNAc residue is indicated in three moieties, reduced glucosamine ($\mathrm{GlcN}^{\mathrm{Red}}$), the acetyl group (Ac), and the D-lactoyl group. Labeled and unlabeled moieties are represented in red and purple, respectively. The fragmentation led to two series of fragments specific of the isotopomers containing the unlabeled glucosamine moiety in the GlcNAc (h1G) or $\mathrm{MurNAc}^{\mathrm{Red}}$ (h1M) residues, respectively (discriminatory fragments). The fragments specific of h1G and of h1M are highlighted by orange and green dots in the mass spectrum. The other fragments are common to the fragmentation patterns of h1G and of h1M. The corresponding peaks are highlighted by blue dots in the mass spectrum. The mass spectrum indicates the presence of both isotopomers as expected from the recycling and synthesis pathways since UDP-MurNAc exclusively derives from UDP-GlcNAc. In the mass spectrum, peaks differing by the loss of $H_2$0 are connected by a dashed line. The tables only contain the m/z values for the fragments containing $H_2$0.An interactive report of the MS^2 analysis is available in Supplementary File F2.2
.

Precursor ion (MS1)	m/z_{obs}	m/z_{calc}	ppm
GlcN(-Ac)-GlcN^{Red}(-Ac)-Lac-Ala-Glu-DAP-Ala	949.429	949.433	-4.0
GlcN(-Ac)-GlcN^{Red}(-Ac)-Lac-Ala-Glu-DAP-Ala	949.429	949.433	-4.0

Discriminatory product ions	m/z_{obs}	m/z_{calc}	ppm	Intensity (a.u.)	Isotopologue
GlcN^{Red}(-Ac)-Lac-Ala-Glu-DAP-Ala	746.351	746.353	3.3	5188	h1M
GlcN^{Red}(-Ac)-Lac-Ala-Glu-DAP-Ala	739.333	739.336	4.7	3690	h1G
GlcN^{Red}(-Ac)-Lac-Ala-Glu-DAP	657.302	657.306	5.2	477	h1M
GlcN^{Red}(-Ac)-Lac-Ala-Glu-DAP	650.287	650.288	2.7	427	h1G
GlcN^{Red}(-Ac)-Lac-Ala-Glu	485.216	485.221	10.3	417	h1M
GlcN^{Red}(-Ac)-Lac-Ala-Glu	478.202	478.204	3.3	210	h1G
GlcN^{Red}(-Ac)-Lac-Ala	356.177	356.178	3.6	1850	h1M
GlcN^{Red}(-Ac)-Lac-Ala	349.158	349.161	8.1	2036	h1G
GlcN^{Red}(-Ac)-Lac	285.140	285.141	4.9	586	h1M
GlcN^{Red}(-Ac)-Lac	278.123	278.124	4.0	622	h1G
GlcN(-Ac)	204.086	204.087	7.2	137	h1M

Common product ion	m/z_{obs}	m/z_{calc}	ppm	Intensity (a.u.)
GlcN(-Ac)-GlcN^{Red}(-Ac)-Lac-Ala-Glu-DAP-Ala	949.433	949.433	0.1	241
GlcN(-Ac)-GlcN^{Red}(-Ac)-Lac-Ala-Glu-DAP-Ala				
Ala-Glu-DAP-Ala	462.217	462.220	6.1	1977
Glu-DAP-Ala	391.180	391.183	6.2	7517
Ala-Glu-DAP	373.172	373.172	1.7	2756
Glu-DAP	302.135	302.135	0.9	7122
DAP-Ala	262.138	262.140	9.0	5564
DAP	173.093	173.093	0.3	796

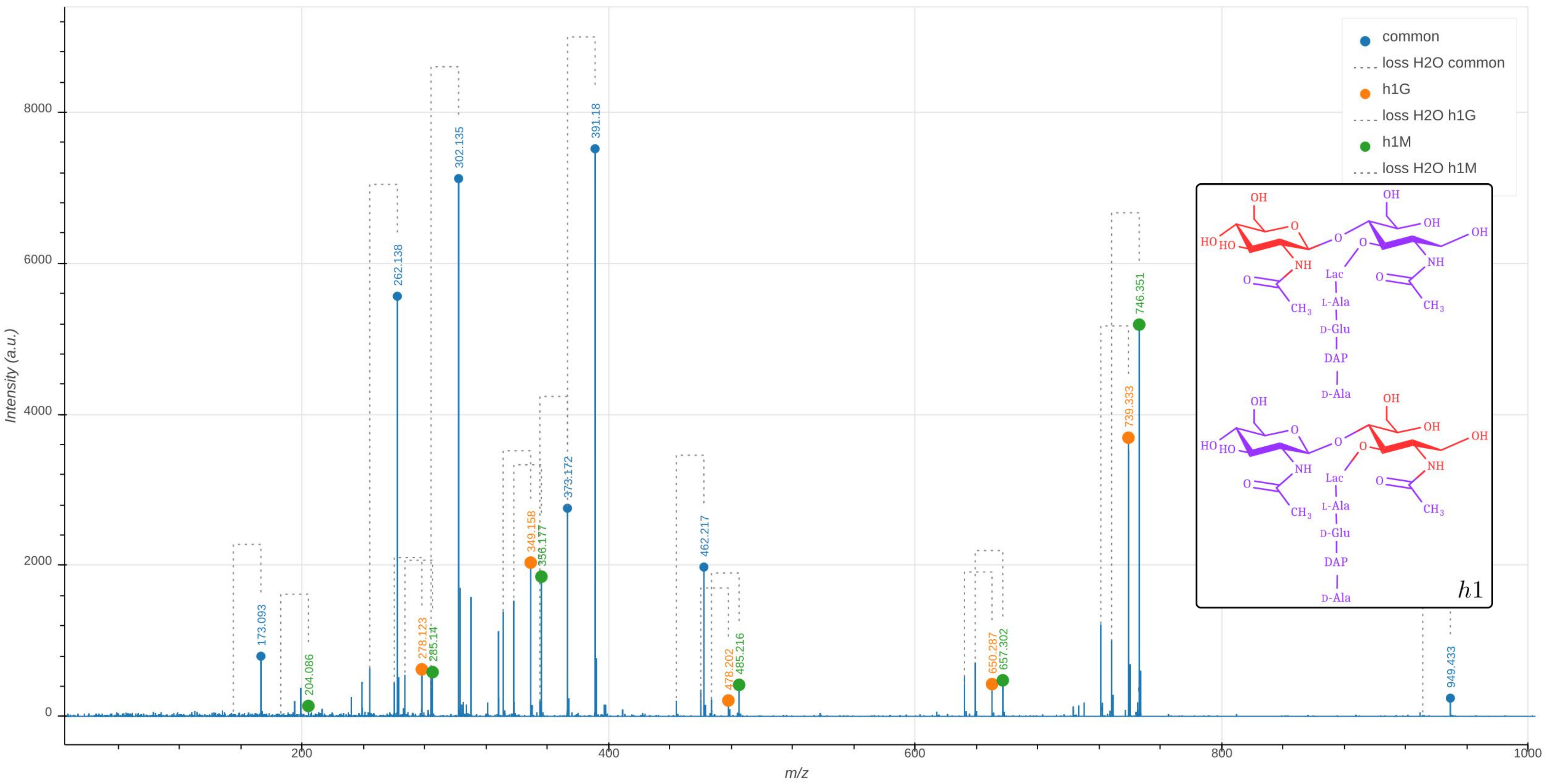

common
loss H2O common
h1G
loss H2O h1G
h1M
loss H2O h1M
Intensity (a.u.)
m/z
173.093
204.086
262.138
278.123
285.14
302.135
349.158
356.177
373.172
391.18
462.217
478.202
485.216
650.287
657.302
739.333
746.351
949.433
Lac
L-Ala
D-Glu
DAP
D-Ala
h1

Supplementary Data 2.3: Tandem mass spectrometry analysis of the h2-type hybrid of the disaccharide-tetrapeptide. The observed m/z_{obs} and calculated m/z_{cal} values of the parental ion, $[\mathrm{M{+}H}]^{+}$, as determined in the absence of fragmentation (MS1), are indicated. The m/z_{calc} value was used to select the ion for fragmentation. The difference between the m/z_{obs} and m/z_{calc} values is indicated in ppm. The GlcNAc residue is indicated in two moieties, glucosamine (GlcN) and the acetyl group (Ac), since these moieties are potentially differentially labeled. For the same reason, the reduced (Red) MurNAc residue is indicated in three moieties, reduced glucosamine ($\mathrm{GlcN^{Red}}$), the acetyl group (Ac), and the D-lactoyl group. Labeled and unlabeled moieties are represented in red and purple, respectively. The peaks corresponding to the list of fragments appearing in the table are highlighted by orange dots in the mass spectrum. In the mass spectrum, peaks differing by the loss of H_20 are connected by a dashed line. The tables only contain the m/z values for the fragments containing H_20.
An interactive report of the MS^2 analysis is available in Supplementary File F2.3 .

Precursor ion (MS1)	m/z_{obs}	m/z_{calc}	ppm
GlcN(-Ac)-$\mathrm{GlcN^{Red}}$(-Ac)-Lac-Ala-Glu-DAP-Ala	961.451	961.454	-2.7

Product ion	m/z_{obs}	m/z_{calc}	ppm	Intensity (a.u.)	Isotopologue
GlcN(-Ac)-$\mathrm{GlcN^{Red}}$(-Ac)-Lac-Ala-Glu-DAP-Ala	961.456	961.454	-2.4	250	h2
$\mathrm{GlcN^{Red}}$(-Ac)-Lac-Ala-Glu-DAP-Ala	758.374	758.375	1.0	15348	h2
$\mathrm{GlcN^{Red}}$(-Ac)-Lac-Ala-Glu-DAP	669.327	669.327	-0.8	1466	h2
GlcN(-Ac)-$\mathrm{GlcN^{Red}}$(-Ac)-Lac-Ala	556.244	556.248	7.1	191	h2
Lac-Ala-Glu-DAP-Ala	553.280	553.280	-0.4	302	h2
$\mathrm{GlcN^{Red}}$(-Ac)-Lac-Ala-Glu	488.224	488.225	1.8	1041	h2
Ala-Glu-DAP-Ala	481.259	481.258	-1.0	3833	h2
Lac-Ala-Glu-DAP	464.235	464.232	-6.3	133	h2
Glu-DAP-Ala	406.212	406.214	6.4	13380	h2
Ala-Glu-DAP	392.208	392.211	6.8	4340	h2
$\mathrm{GlcN^{Red}}$(-Ac)-Lac-Ala	353.167	353.168	3.4	6673	h2
Glu-DAP	317.165	317.167	3.8	12830	h2
$\mathrm{GlcN^{Red}}$(-Ac)-Lac	278.122	278.124	7.6	1991	h2
DAP-Ala	271.156	271.158	7.2	10798	h2
$\mathrm{GlcN^{Red}}$(-Ac)	206.103	206.103	-2.8	163	h2
GlcN(-Ac)	204.085	204.087	12.7	229	h2
DAP	182.108	182.110	9.5	1297	h2

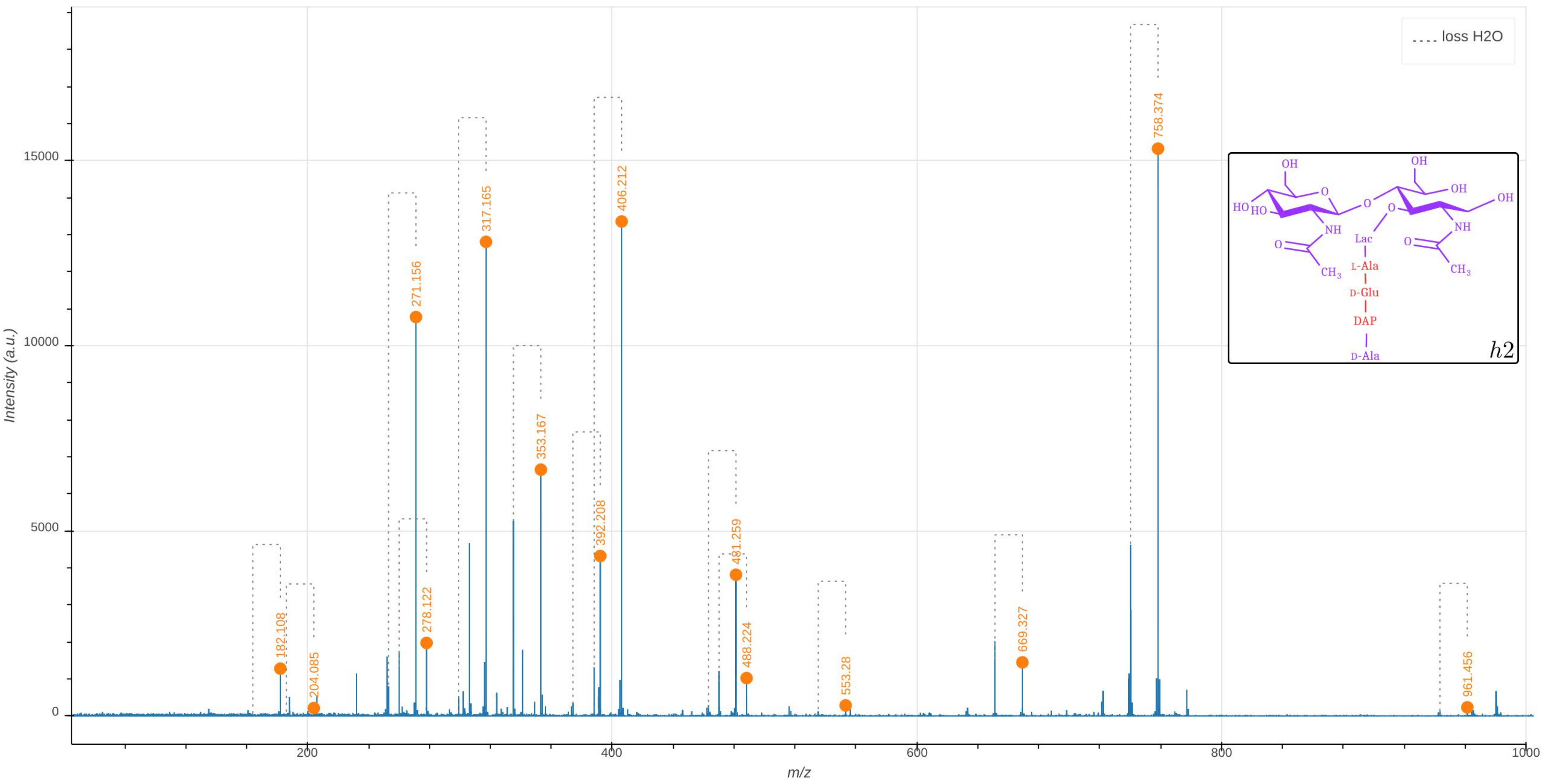

loss H2O
Intensity (a.u.)
m/z
0
5000
10000
15000
200
400
600
800
1000
182.108
204.085
271.156
278.122
317.165
353.167
392.208
406.212
481.259
488.224
553.28
669.327
758.374
961.456
OH
HO
HO
O
NH
O
CH3
Lac
L-Ala
D-Glu
DAP
D-Ala
OH
OH
O
OH
NH
O
CH3
h2

Supplementary Data 2.4: Tandem mass spectrometry analysis of the h3-type hybrid of the disaccharide-tetrapeptide. The observed m/z_{obs} and calculated m/z_{cal} values of the parental ion, $[\text{M+H}]^+$, as determined in the absence of fragmentation (MS1), are indicated. The m/z_{calc} value was used to select the ion for fragmentation. The difference between the m/z_{obs} and m/z_{calc} values is indicated in ppm. The GlcNAc residue is indicated in two moieties, glucosamine (GlcN) and the acetyl group (Ac), since these moieties are potentially differentially labeled. For the same reason, the reduced (Red) MurNAc residue is indicated in three moieties, reduced glucosamine (GlcN^{Red}), the acetyl group (Ac), and the D-lactoyl group. Labeled and unlabeled moieties are represented in red and purple, respectively. The peaks corresponding to the list of fragments appearing in the table are highlighted by orange dots in the mass spectrum. In the mass spectrum, peaks differing by the loss of H_20 are connected by a dashed line. The tables only contain the m/z values for the fragments containing H_20. The presence of the h3 isotopomer is expected since cells of *E. coli* contain the main PG precursor, UDP-MurNAc-pentapeptide in considerable amounts (about 2% compared to the total amount of dissaccharide peptides in the cell wall). An interactive report of the MS^2 analysis is available in Supplementary File F2.4 .

Precursor ion (MS1)	m/z_{obs}	m/z_{calc}	ppm
GlcN(-Ac)-GlcN^{Red}(-Ac)-Lac-Ala-Glu-DAP-Ala	977.490	977.49	-5.0

Product ion	m/z_{obs}	m/z_{calc}	ppm	Intensity (a.u.)	Isotopologue
GlcN^{Red}(-Ac)-Lac-Ala-Glu-DAP-Ala	774.413	774.416	3.0	3734	h3
GlcN^{Red}(-Ac)-Lac-Ala-Glu-DAP	681.368	681.361	-9.8	306	h3
GlcN^{Red}(-Ac)-Lac-Ala-Glu	500.260	500.259	-2.0	259	h3
Ala-Glu-DAP-Ala	485.265	485.266	1.6	834	h3
Glu-DAP-Ala	410.220	410.221	3.9	2916	h3
Ala-Glu-DAP	392.208	392.211	6.4	1481	h3
GlcN^{Red}(-Ac)-Lac-Ala	365.199	365.202	7.3	1567	h3
Glu-DAP	317.165	317.167	3.4	3046	h3
GlcN^{Red}(-Ac)-Lac	290.156	290.158	7.5	508	h3
DAP-Ala	275.163	275.165	6.7	2174	h3
DAP	182.110	182.110	0.1	354	h3

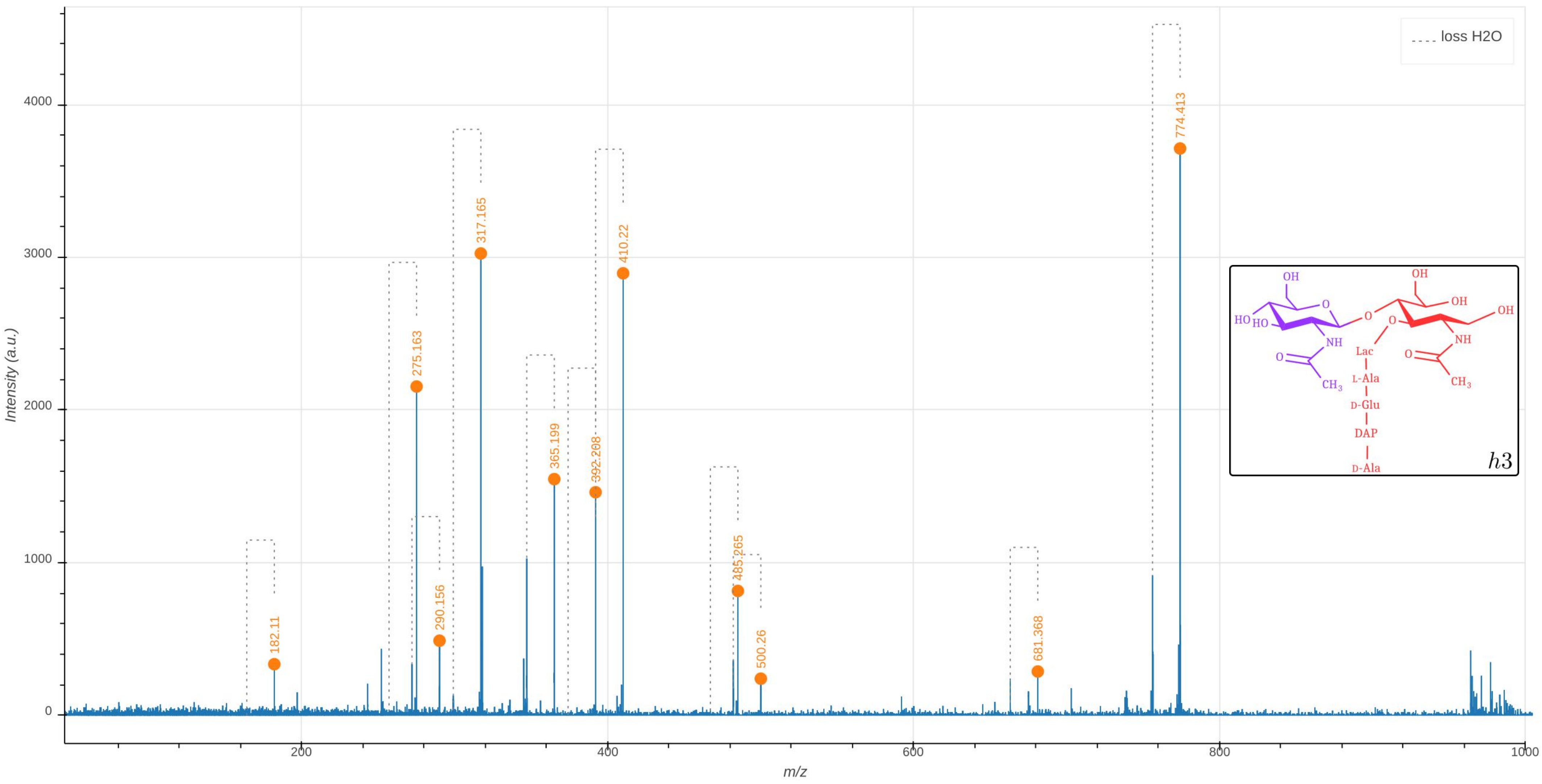

loss H2O
4000
3000
2000
1000
0
Intensity (a.u.)
200
400
600
800
1000
m/z
182.11
275.163
290.156
317.165
365.199
392.208
410.22
485.265
500.26
681.368
774.413
OH
HO
HO
O
NH
O
CH3
O
OH
OH
OH
O
NH
O
CH3
Lac
L-Ala
D-Glu
DAP
D-Ala
h3

Additional Monomer hybrids

Supplementary Data 2.5: Tandem mass spectrometry analysis of the hAla-type hybrid of the disaccharide-tetrapeptide. The observed m/z_{obs} and calculated m/z_{cal} values of the parental ion, $[M+H]^+$, as determined in the absence of fragmentation (MS1), are indicated. The m/z_{calc} value was used to select the ion for fragmentation. The difference between the m/z_{obs} and m/z_{calc} values is indicated in ppm. The GlcNAc residue is indicated in two moieties, glucosamine (GlcN) and the acetyl group (Ac), since these moieties are potentially differentially labeled. For the same reason, the reduced (Red) MurNAc residue is indicated in three moieties, reduced glucosamine ($\mathrm{GlcN}^{\mathrm{Red}}$), the acetyl group (Ac), and the D-lactoyl group. Labeled and unlabeled moieties are represented in red and purple, respectively. The peaks corresponding to the list of fragments appearing in the table are highlighted by orange dots in the mass spectrum. In the mass spectrum, peaks differing by the loss of H_20 are connected by a dashed line. The tables only contain the m/z values for the fragments containing H_20.
An interactive report of the MS2 analysis is available in Supplementary File F2.5.

Parent (MS1)	m/z_{obs}	m/z_{calc}	ppm
GlcN(-Ac)-GlcN^{Red}(-Ac)-Lac-Ala-Glu-DAP-Ala	946.420	946.423	-2.9

Fragment	m/z_{obs}	m/z_{calc}	ppm	Intensity (a.u.)	Isotopologue
GlcN(-Ac)-GlcN^{Red}(-Ac)-Lac-Ala-Glu-DAP-Ala	946.414	946.423	8.8	227	hAla4
GlcN^{Red}(-Ac)-Lac-Ala-Glu-DAP-Ala	743.340	743.343	4.3	1657	hAla4
Ala-Glu-DAP-Ala	466.232	466.227	-9.5	194	hAla4
Glu-DAP-Ala	395.191	395.190	-3.2	616	hAla4
Ala-Glu-DAP	373.172	373.172	0.3	84	hAla4
GlcN^{Red}(-Ac)-Lac-Ala	349.162	349.161	-2.2	131	hAla4
Glu-DAP	302.133	302.135	7.2	279	hAla4
GlcN^{Red}(-Ac)-Lac	278.125	278.124	-2.5	60	hAla4
DAP-Ala	266.146	266.147	4.3	73	hAla4
DAP	173.091	173.093	10.9	150	hAla4

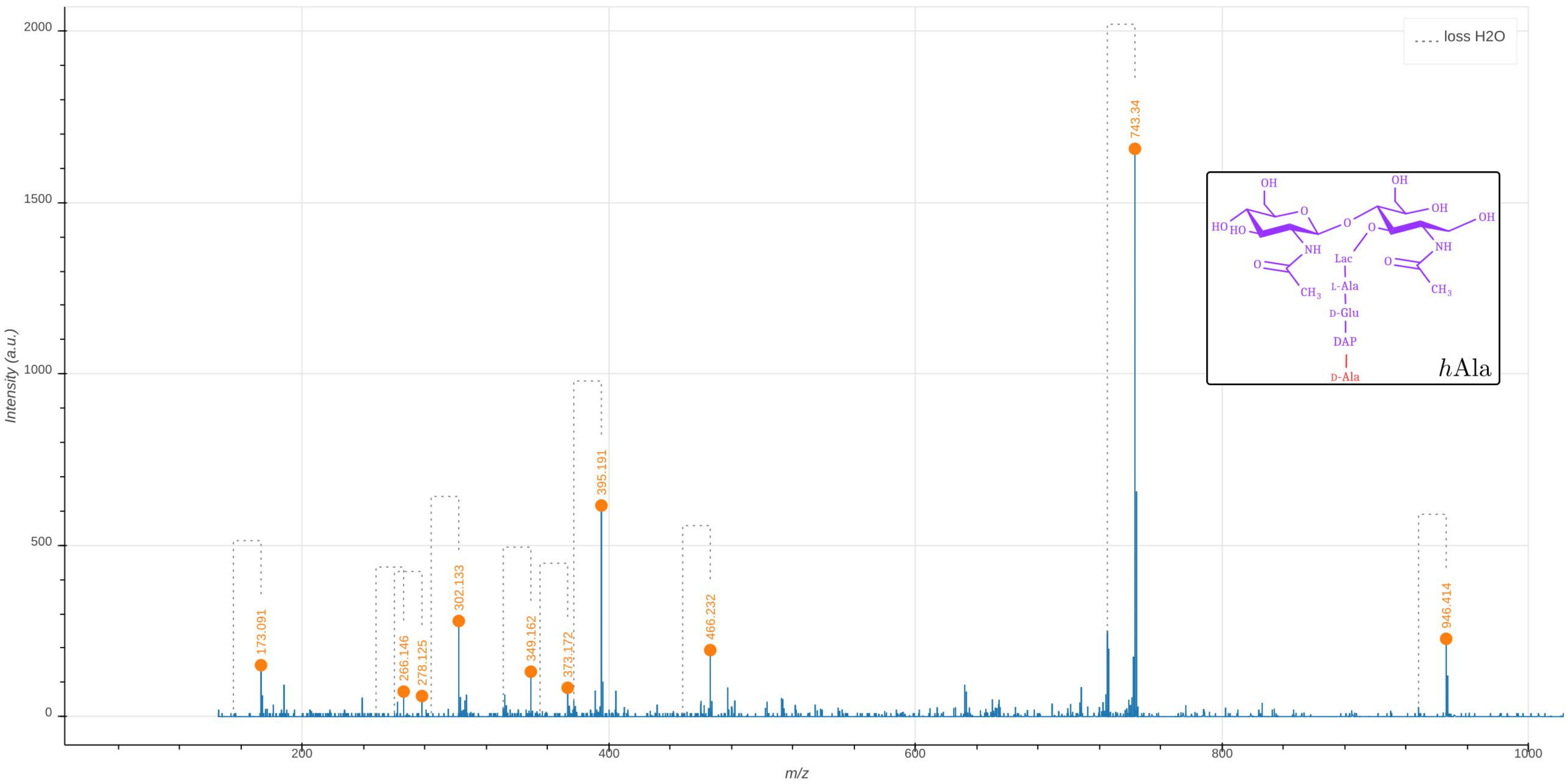

loss H2O
743.34
395.191
302.133
173.091
266.146
278.125
349.162
373.172
466.232
946.414
Intensity (a.u.)
m/z
OH
HO
HO
O
NH
O
CH3
Lac
L-Ala
D-Glu
DAP
D-Ala
OH
OH
OH
hAla

Supplementary Data 2.6: Tandem mass spectrometry analysis of the h1h2-type hybrid of the disaccharide-tetrapeptide. The molecule is fully unlabeled except for one of the two glucosamine moieties present in GlcNAc or MurNAc leading to the presence of two isotopomers.

The observed m/z_{obs} and calculated m/z_{calc} values of the parental ion, $[\mathrm{M+H}]^+$, as determined in the absence of fragmentation (MS^1), are indicated. The m/z_{cal} value was used to select the ion for fragmentation. The difference between the m/z_{obs} and m/z_{cal} values is indicated in ppm. The GlcNAc residue is indicated in two moieties, glucosamine (GlcN) and the acetyl group (Ac), since these moieties are potentially differentially labeled. For the same reason, the reduced (Red) MurNAc residue is indicated in three moieties, reduced glucosamine ($\mathrm{GlcN^{Red}}$), the acetyl group (Ac), and the D-lactoyl group. Labeled and unlabeled moieties are represented in red and purple, respectively. The fragmentation led to two series of fragments specific of the isotopomers containing the unlabeled glucosamine moiety in the GlcNAc (h1Gh2) or $\mathrm{MurNAc^{Red}}$ (h1Mh2) residues, respectively (discriminatory fragments). The fragments specific of h1Gh2 and of h1Mh2 are highlighted by orange and green dots in the mass spectrum. The other fragments are common to the fragmentation patterns of h1Gh2 and of h1Mh2. The corresponding peaks are highlighted by blue dots in the mass spectrum. The mass spectrum indicates the presence of both isotopomers as expected from the recycling and synthesis pathways since UDP-MurNAc exclusively derives from UDP-GlcNAc. In the mass spectrum, peaks differing by the loss of H_20 are connected by a dashed line. The tables only contain the m/z values for the fragments containing H_20. An interactive report of the MS2 analysis is available in Supplementary File F2.6.

Precursor ion (MS1)	m/z_{obs}	m/z_{calc}	ppm
GlcN(-Ac)-GlcN^{Red}(-Ac)-Lac-Ala-Glu-DAP-Ala	968.471	968.471	0.2
GlcN(-Ac)-GlcN^{Red}(-Ac)-Lac-Ala-Glu-DAP-Ala	968.471	968.471	0.2

Discriminatory product ions	m/z_{obs}	m/z_{calc}	ppm	Intensity (a.u.)	Isotopologue
GlcN^{Red}(-Ac)-Lac-Ala-Glu-DAP-Ala	765.390	765.392	2.2	469	h1Mh2
GlcN^{Red}(-Ac)-Lac-Ala-Glu-DAP-Ala	758.370	758.375	6.2	309	h1Gh2

Common product ions	m/z_{obs}	m/z_{calc}	ppm	Intensity (a.u.)
Glu-DAP-Ala	406.212	406.214	5.8	151
Glu-DAP	317.163	317.167	12.6	121
DAP	182.108	182.110	11.6	60

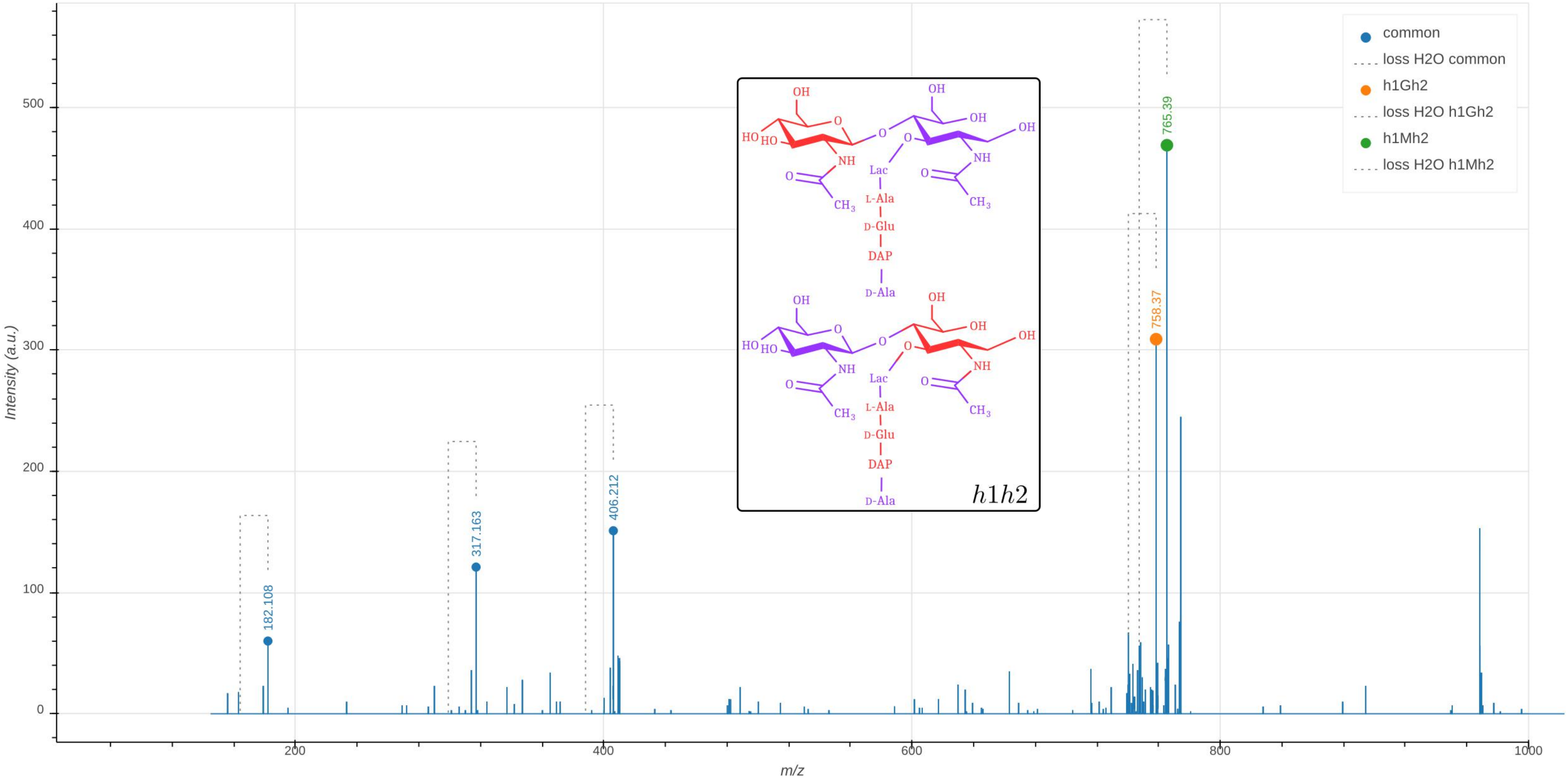
Intensity (a.u.)
m/z
common
loss H2O common
h1Gh2
loss H2O h1Gh2
h1Mh2
loss H2O h1Mh2
182.108
317.163
406.212
758.37
765.39
Lac
L-Ala
D-Glu
DAP
D-Ala
h1h2

Supplementary Data 2.7: Tandem mass spectrometry analysis of the h3Ala-type hybrid of the disaccharide-tetrapeptide. The observed m/z_{obs} and calculated m/z_{cal} values of the parental ion, $[M+H]^+$, as determined in the absence of fragmentation (MS1), are indicated. The m/z_{calc} value was used to select the ion for fragmentation. The difference between the m/z_{obs} and m/z_{calc} values is indicated in ppm. The GlcNAc residue is indicated in two moieties, glucosamine (GlcN) and the acetyl group (Ac), since these moieties are potentially differentially labeled. For the same reason, the reduced (Red) MurNAc residue is indicated in three moieties, reduced glucosamine (GlcN^{Red}), the acetyl group (Ac), and the D-lactoyl group. Labeled and unlabeled moieties are represented in red and purple, respectively. The peaks corresponding to the list of fragments appearing in the table are highlighted by orange dots in the mass spectrum. In the mass spectrum, peaks differing by the loss of H_20 are connected by a dashed line. The tables only contain the m/z values for the fragments containing H_20.
An interactive report of the MS2 analysis is available in Supplementary File F2.7.

Precursor Ion (MS1)	m/z_{obs}	m/z_{calc}	ppm
GlcN(-Ac)-GlcN^{Red}(-Ac)-Lac-Ala-Glu-DAP-Ala	973.484	973.488	-3.9

Product ion	m/z_{obs}	m/z_{calc}	ppm	Intensity (a.u.)
GlcN(-Ac)-GlcN^{Red}(-Ac)-Lac-Ala-Glu-DAP-Ala	973.492	973.488	-3.7	161
GlcN^{Red}(-Ac)-Lac-Ala-Glu-DAP-Ala	770.407	770.409	2.1	2018
GlcN^{Red}(-Ac)-Lac-Ala-Glu-DAP	681.362	681.361	-1.4	91
GlcN^{Red}(-Ac)-Lac-Ala-Glu	500.260	500.259	-3.4	65
Ala-Glu-DAP-Ala	481.254	481.258	9.6	113
Glu-DAP-Ala	406.215	406.214	-2.1	544
Ala-Glu-DAP	392.207	392.211	10.6	138
GlcN^{Red}(-Ac)-Lac-Ala	365.201	365.202	2.0	332
Glu-DAP	317.167	317.167	-1.6	259
GlcN^{Red}(-Ac)-Lac	290.158	290.158	-0.3	62
DAP-Ala	271.155	271.158	11.1	111
DAP	182.108	182.110	12.8	20

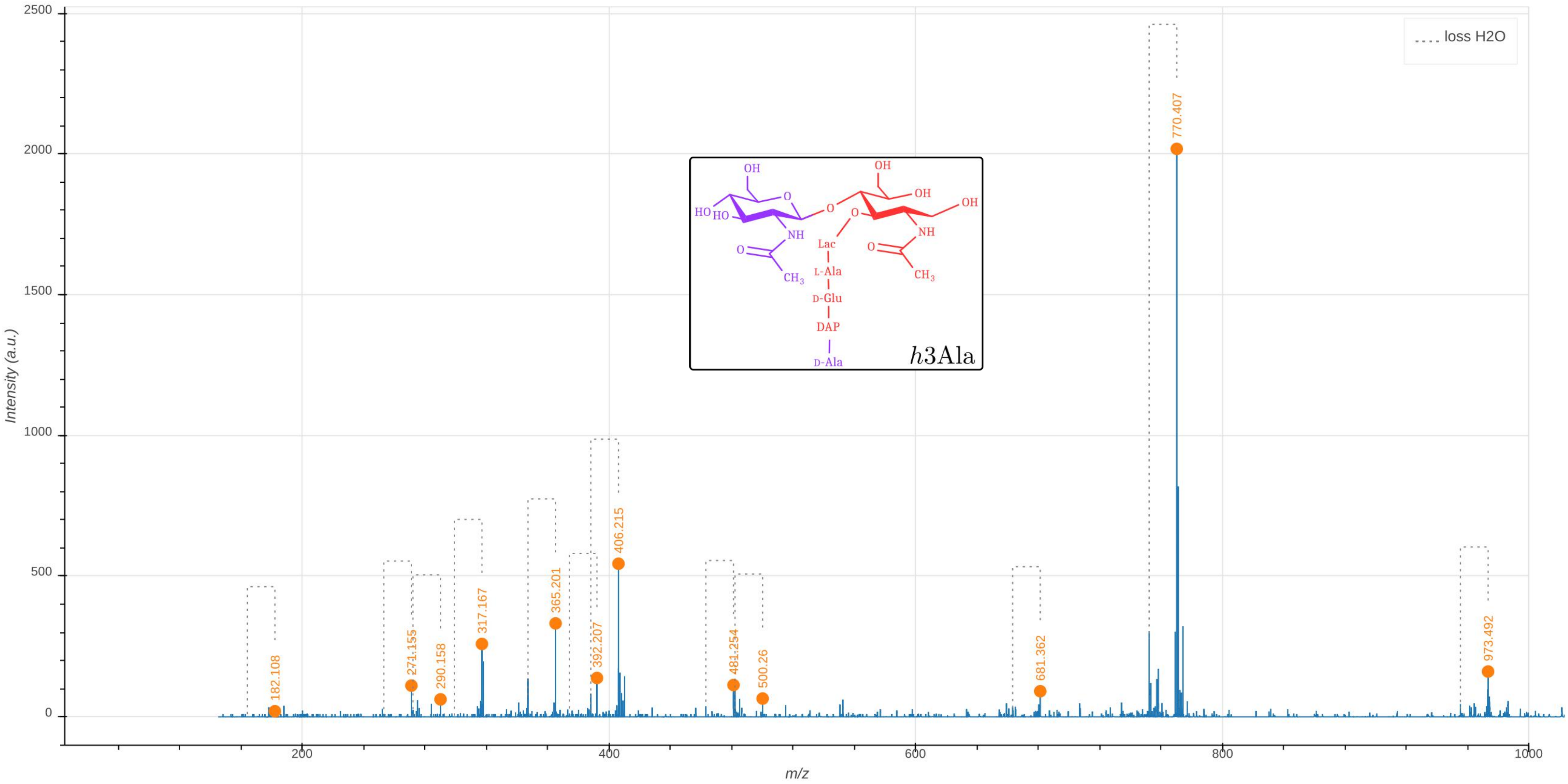

loss H2O
770.407
406.215
365.201
317.167
392.207
271.155
290.158
182.108
481.254
500.26
681.362
973.492
OH
O
HO
HO
NH
O
CH3
OH
OH
OH
O
O
NH
O
CH3
Lac
L-Ala
D-Glu
DAP
D-Ala
h3Ala
Intensity (a.u.)
m/z
0
500
1000
1500
2000
2500
200
400
600
800
1000

A - GM-Tripeptide

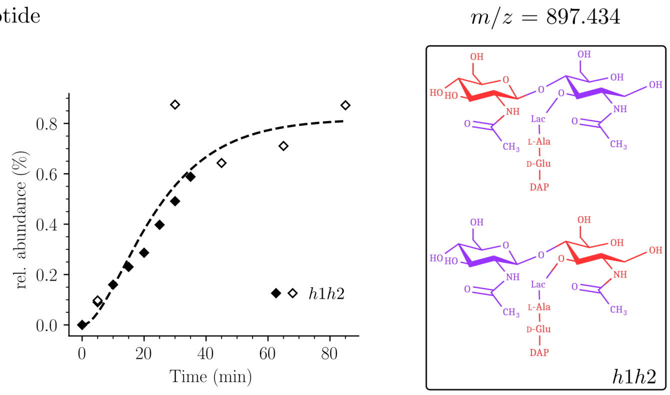

B - GM-Tetrapeptide

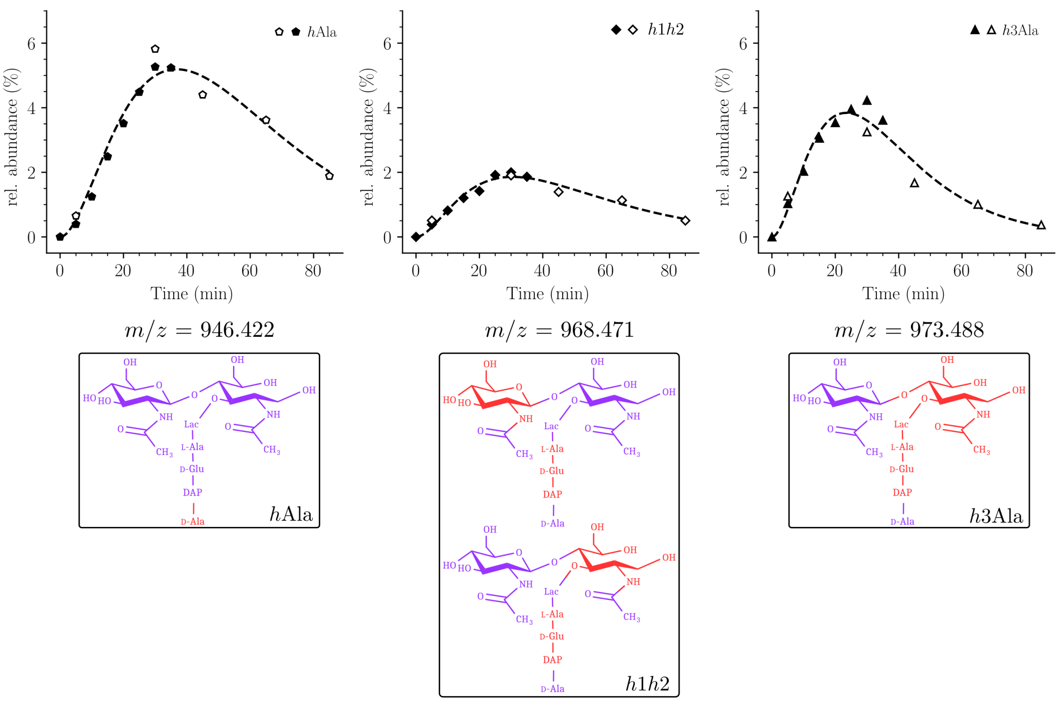

Supplementary Data 2.8: Timecourses and structures of additional monomer hybrids. (**A**) Structure and timecourse of the *h*1*h*2 hybrid of the disaccharide tripeptide monomer combining a recycled tripeptide stem (*h*2) with a recycled glucosamine moeity (*h*1). (**B**) Additional hybrids of the disaccharide tetrapeptide comprise *h*Ala (labeled C-terminal D-Ala^4), *h*1*h*2 (see (**A**)) and *h*3Ala (neo-synthesiszed GlcNAc and C-terminal D-Ala^4) Since disaccharide tripeptides are issued from disaccharide tetrapeptides by removal of the C-terminal D-Ala^4, *h*Ala and *h*3Ala are not detected for the tripeptide as these are converted into the uniformly unlabeled and *h*3 hybrid of the tripeptide, respectively. For structural characterization of the *h*Ala, *h*1*h*2, and *h*3Ala hybrids see Supplementary Data above.

Dimers

Tri-Tri

Supplementary Data 3.1: Tandem mass spectrometry analysis of the uniformly labeled Tri(3→3)Tri dimer. The observed m/z_{obs} and calculated m/z_{cal} values of the parental ion, $[\mathrm{M+2H}]^{2+}$, as determined in the absence of fragmentation (MS1), are indicated. The m/z_{calc} value was used to select the ion for fragmentation. The difference between the m/z_{obs} and m/z_{calc} values is indicated in ppm. The GlcNAc residue is indicated in two moieties, glucosamine (GlcN) and the acetyl group (Ac), since these moieties are potentially differentially labeled. For the same reason, the reduced (Red) MurNAc residue is indicated in three moieties, reduced glucosamine ($\mathrm{GlcN^{Red}}$), the acetyl group (Ac), and the D-lactoyl group. Labeled and unlabeled moieties are represented in red and purple, respectively. The peaks corresponding to the list of fragments appearing in the table are highlighted by orange dots in the mass spectrum. In the mass spectrum, peaks differing by the loss of H_20 are connected by a dashed line. The tables only contain the m/z values for the fragments containing H_20. The arrows indicate the position of the 3→3 and 4→3 crosslinks. Residues in the acceptor are underlined.
An interactive report of the MS2 analysis is available in Supplementary File F3.1.

Precursor ion (MS1)	m/z_{obs} $[\mathrm{M+2H}]^{2+}$	m/z_{calc} $[\mathrm{M+2H}]^{2+}$	ppm
GlcN(-Ac)-GlcN^{Red}(-Ac)-Lac-Ala-Glu-DAP→DAP-Glu-Ala-Lac-GlcN^{Red}(-Ac)-GlcN(-Ac)	902.464	902.469	-5.9

Product ion	m/z_{obs} $[\mathrm{M+H}]^{1+}$	m/z_{calc} $[\mathrm{M+H}]^{1+}$	ppm	Intensity (a.u.)
GlcN(-Ac)-GlcN^{Red}(-Ac)-Lac-Ala-Glu-DAP→DAP-Glu-Ala-Lac-GlcN^{Red}(-Ac)	1591.833	1591.828	-3.4	52
GlcN^{Red}(-Ac)-Lac-Ala-Glu-DAP→DAP-Glu-Ala-Lac-GlcN^{Red}(-Ac)	1379.721	1379.724	2.3	709
GlcN^{Red}(-Ac)-Lac-Ala-Glu-DAP→DAP-Glu-Ala	1090.570	1090.574	3.7	53
GlcN^{Red}(-Ac)-Lac-Ala-Glu-DAP→DAP-Glu	1015.523	1015.530	7.4	133
DAP-Glu-Ala-Lac-GlcN^{Red}(-Ac)	699.371	699.371	1.1	71
GlcN^{Red}(-Ac)-Lac-Ala-Glu-DAP	681.356	681.361	6.9	286
GlcN^{Red}(-Ac)-Lac-Ala	365.200	365.202	5.6	77
GlcN(-Ac)	213.109	213.111	7.8	860

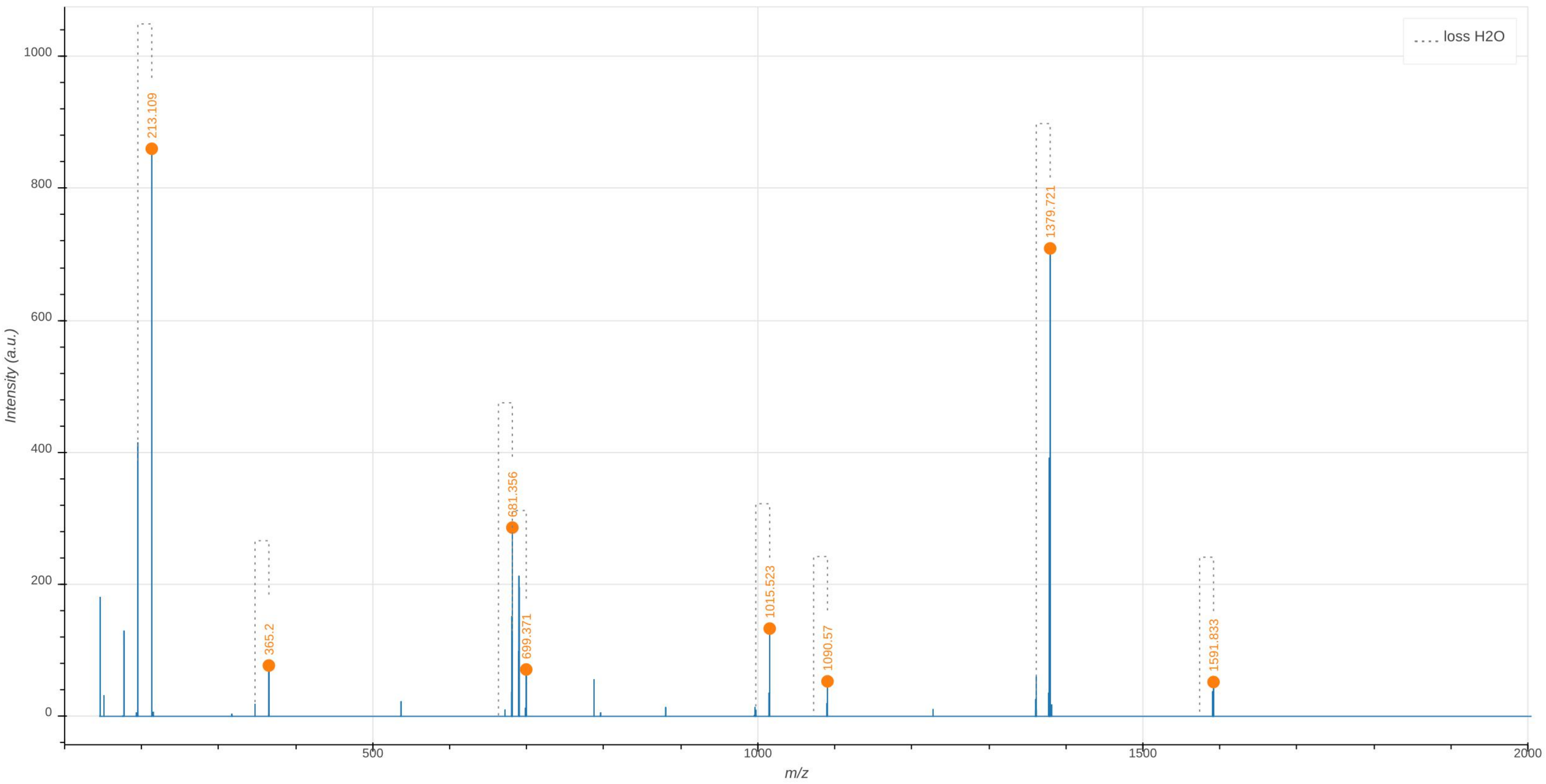

loss H2O
213.109
365.2
681.356
699.371
1015.523
1090.57
1379.721
1591.833
Intensity (a.u.)
m/z
0
200
400
600
800
1000
500
1000
1500
2000

Supplementary Data 3.2: Tandem mass spectrometry analysis of the uniformly unlabeled Tri(3→3)Tri dimer. The observed m/z_{obs} and calculated m/z_{cal} values of the parental ion, $[M+2H]^{2+}$, as determined in the absence of fragmentation (MS1), are indicated. The m/z_{calc} value was used to select the ion for fragmentation. The difference between the m/z_{obs} and m/z_{calc} values is indicated in ppm. The GlcNAc residue is indicated in two moieties, glucosamine (GlcN) and the acetyl group (Ac), since these moieties are potentially differentially labeled. For the same reason, the reduced (Red) MurNAc residue is indicated in three moieties, reduced glucosamine (GlcN^{Red}), the acetyl group (Ac), and the D-lactoyl group. Labeled and unlabeled moieties are represented in red and purple, respectively. The peaks corresponding to the list of fragments appearing in the table are highlighted by orange dots in the mass spectrum. In the mass spectrum, peaks differing by the loss of H_20 are connected by a dashed line. The tables only contain the m/z values for the fragments containing H_20.
The arrows indicate the position of the 3→3 and 4→3 crosslinks. Residues in the acceptor are underlined.
An interactive report of the MS2 analysis is available in Supplementary File F3.2.

Precursor ion (MS1)	m/z_{obs} $[M+2H]^{2+}$	m/z_{calc} $[M+2H]^{2+}$	ppm
GlcN(-Ac)-GlcN^{Red}(-Ac)-Lac-Ala-Glu-DAP→DAP-Glu-Ala-Lac-GlcN^{Red}(-Ac)-GlcN(-Ac)	862.373	862.373	0.0

Product ion	m/z_{obs} $[M+H]^{1+}$	m/z_{calc} $[M+H]^{1+}$	ppm	Intensity (a.u.)
GlcN(-Ac)-GlcN^{Red}(-Ac)-Lac-Ala-Glu-DAP→DAP-Glu-Ala-Lac-GlcN^{Red}(-Ac)	1520.658	1520.659	0.9	528
GlcN^{Red}(-Ac)-Lac-Ala-Glu-DAP→DAP-Glu-Ala-Lac-GlcN^{Red}(-Ac)	1317.581	1317.580	-1.3	14608
GlcN(-Ac)-GlcN^{Red}(-Ac)-Lac-Ala-Glu-DAP→DAP-Glu	1172.522	1172.506	-13.7	195
GlcN^{Red}(-Ac)-Lac-Ala-Glu-DAP→DAP-Glu-Ala-Lac	1112.491	1112.485	-5.3	340
GlcN^{Red}(-Ac)-Lac-Ala-Glu-DAP→DAP-Glu-Ala	1040.466	1040.464	-2.0	2012
GlcN^{Red}(-Ac)-Lac-Ala-Glu-DAP→DAP-Glu	969.428	969.426	-1.3	10207
Lac-Ala-Glu-DAP→DAP-Glu-Ala-Lac	907.403	907.390	-14.5	267
GlcN^{Red}(-Ac)-Lac-Ala-Glu-DAP→DAP	840.384	840.384	-0.5	4637
Lac-Ala-Glu-DAP→DAP-Glu-Ala	835.377	835.369	-10.5	208
Lac-Ala-Glu-DAP→DAP-Glu	764.336	764.331	-5.8	517
Ala-Glu-DAP→DAP-Glu-Ala	763.357	763.347	-12.3	114
Ala-Glu-DAP→DAP-Glu	692.315	692.310	-6.1	860
DAP-Glu-Ala-Lac-GlcN^{Red}(-Ac)	668.298	668.299	1.6	6970
GlcN^{Red}(-Ac)-Lac-Ala-Glu-DAP	650.291	650.288	-4.1	6495
Lac-Ala-Glu-DAP→DAP	635.289	635.289	-0.5	324
Glu-DAP→DAP-Glu	621.278	621.273	-7.6	1912
Ala-Glu-DAP→DAP	563.264	563.268	5.8	800
Glu-DAP→DAP	492.233	492.231	-4.0	3051
GlcN^{Red}(-Ac)-Lac-Ala-Glu	478.208	478.204	-9.4	449
DAP-Glu-Ala-Lac	463.205	463.204	-2.7	521
DAP-Glu-Ala	391.185	391.183	-5.5	608
Ala-Glu-DAP	373.173	373.172	-0.8	1284
DAP→DAP	363.190	363.188	-4.9	1374
GlcN^{Red}(-Ac)-Lac-Ala	349.162	349.161	-1.6	2576
DAP-Glu	320.146	320.146	-0.4	1973
Glu-DAP	302.136	302.135	-1.6	3703
GlcN^{Red}(-Ac)-Lac	278.126	278.124	-6.0	576

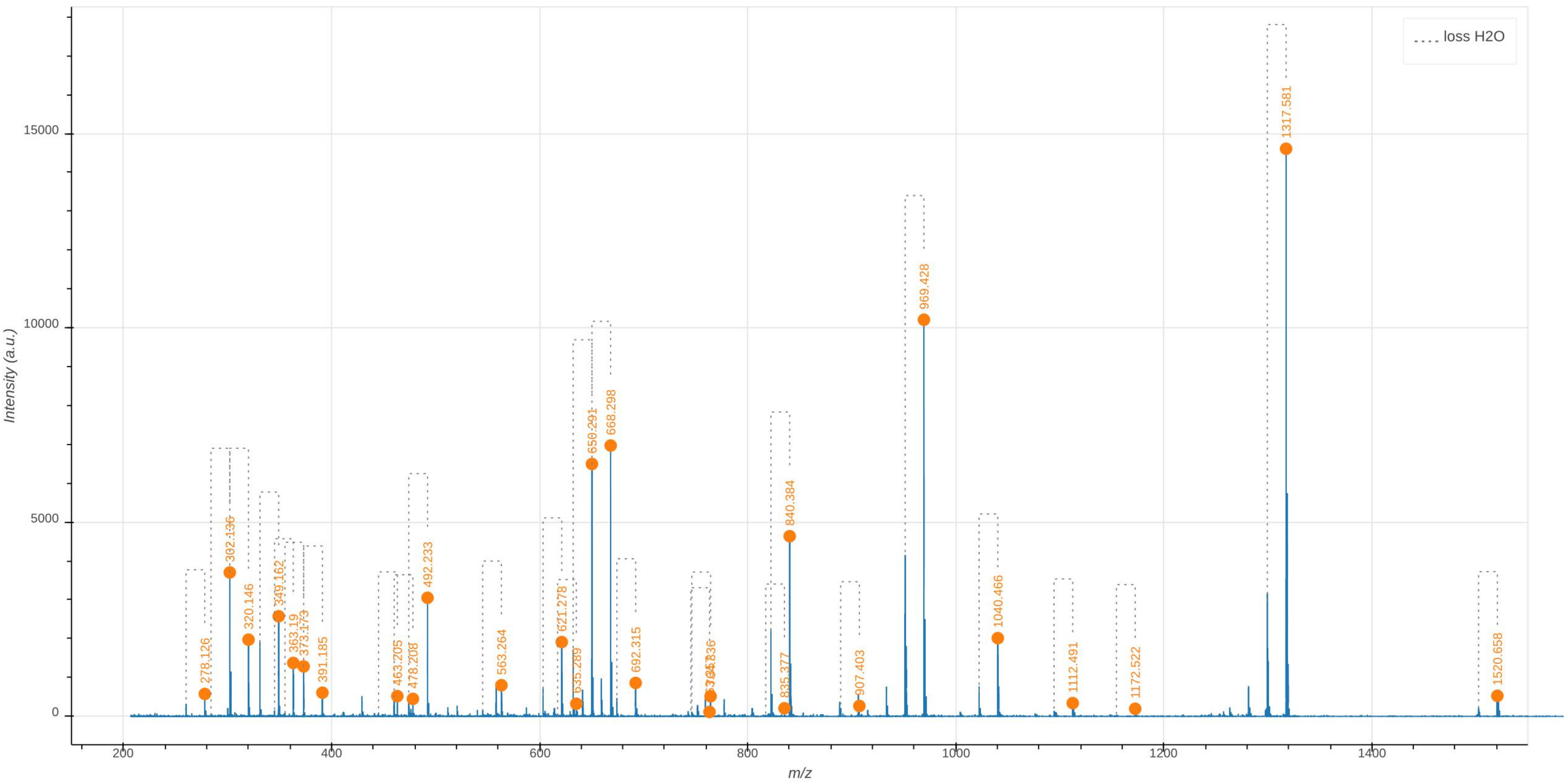

loss H2O
Intensity (a.u.)
m/z
278.126
302.136
320.146
349.162
363.19
373.173
391.185
463.205
478.208
492.233
563.264
621.278
635.289
650.291
668.298
692.315
764.336
835.377
840.384
907.403
969.428
1040.466
1112.491
1172.522
1317.581
1520.658

Supplementary Data 3.3: Tandem mass spectrometry analysis of the Tri→Tri dimer containing a uniformly labeled disaccharide-tripeptide cross-linked to a uniformly unlabeled disaccharide-tripeptide (3→3 cross-link). The observed m/z_{obs} and calculated m/z_{cal} values of the parental ion, $[M+2H]^{2+}$, as determined in the absence of fragmentation (MS1), are indicated. The m/z_{calc} value was used to select the ion for fragmentation. The difference between the m/z_{obs} and m/z_{calc} values is indicated in ppm. The GlcNAc residue is indicated in two moieties, glucosamine (GlcN) and the acetyl group (Ac), since these moieties are potentially differentially labeled. For the same reason, the reduced (Red) MurNAc residue is indicated in three moieties, reduced glucosamine ($GlcN^{Red}$), the acetyl group (Ac), and the D-lactoyl group. Labeled and unlabeled moieties are represented in red and purple, respectively. The fragmentation potentially leads to two series of fragments (discriminatory fragments) specific of the isotopomers containing the fully labeled disaccharide subunit in the donor (all heavy-all light) or acceptor position (all light-all heavy). These fragments are highlighted by green and orange dots in the mass spectrum, respectively. The other fragments are common to the fragmentation of the two isotopomers (highlighted by blue dots in the mass spectrum). The mass spectrum indicates the presence of the all light-all heavy isotopomers (orange dots). The peak at m/z_{obs} 681.358 (green dot) can also be accounted for by the loss of H_20 from the peak at 699.367 indicating that all peaks may be accounted for by the presence of the all light-all heavy isotopomer.

In the mass spectrum, peaks differing by the loss of H_20 are connected by a dashed line. The arrows indicate the position of the 3→3 and 4→3 crosslinks. Residues in the acceptor are underlined.

An interactive report of the MS2 analysis is available in Supplementary File F3.3.

Precursor ion (MS1)	m/z_{obs} $[M+2H]^{2+}$	m/z_{calc} $[M+2H]^{2+}$	ppm
GlcN(-Ac)-GlcN^{Red}(-Ac)-Lac-Ala-Glu-DAP→DAP-Glu-Ala-Lac-GlcN^{Red}(-Ac)-GlcN(-Ac)	882.421	882.421	-0.0
GlcN(-Ac)-GlcN^{Red}(-Ac)-Lac-Ala-Glu-DAP→DAP-Glu-Ala-Lac-GlcN^{Red}(-Ac)-GlcN(-Ac)	882.421	882.421	-0.0

Discriminatory product ions	m/z_{obs} $[M+H]^{1+}$	m/z_{calc} $[M+H]^{1+}$	ppm	Intensity (a.u.)	Isotopologue
DAP-Glu-Ala-Lac-GlcN^{Red}(-Ac)	699.367	699.371	6.5	678	all light-all heavy
GlcN^{Red}(-Ac)-Lac-Ala-Glu-DAP	681.358	681.361	4.8	325	all heavy-all light
DAP-Glu	335.173	335.177	13.8	198	all light-all heavy
Glu-DAP	302.132	302.135	11.5	281	all light-all heavy

Common product ions	m/z_{obs} $[M+H]^{1+}$	m/z_{calc} $[M+H]^{1+}$	ppm	Intensity (a.u.)
GlcN^{Red}(-Ac)-Lac-Ala-Glu-DAP→DAP-Glu-Ala-Lac-GlcN^{Red}(-Ac)	1348.645	1348.652	5.6	912
GlcN^{Red}(-Ac)-Lac-Ala-Glu-DAP→DAP-Glu-Ala-Lac-GlcN^{Red}(-Ac)				
GlcN^{Red}(-Ac)-Lac-Ala-Glu-DAP→DAP-Glu	1000.498	1000.499	0.6	242
Glu-DAP→DAP-Glu-Ala-Lac-GlcN^{Red}(-Ac)				
GlcN^{Red}(-Ac)-Lac-Ala-Glu-DAP→DAP-Glu	984.450	984.458	7.6	428
Glu-DAP→DAP-Glu-Ala-Lac-GlcN^{Red}(-Ac)				
GlcN^{Red}(-Ac)-Lac-Ala-Glu-DAP→DAP	871.456	871.456	0.5	183
DAP→DAP-Glu-Ala-Lac-GlcN^{Red}(-Ac)				
DAP→DAP-Glu-Ala-Lac-GlcN^{Red}(-Ac)	849.398	849.401	3.8	136
GlcN^{Red}(-Ac)-Lac-Ala-Glu-DAP→DAP				
Glu-DAP→DAP-Glu	636.300	636.305	7.0	163
Glu-DAP→DAP-Glu				
DAP→DAP-Glu	507.260	507.262	3.0	190
Glu-DAP→DAP				
DAP→DAP	372.198	372.206	19.4	125
DAP→DAP				
GlcN^{Red}(-Ac)-Lac-Ala	365.199	365.202	9.2	182
Ala-Lac-GlcN^{Red}(-Ac)				
GlcN(-Ac)	213.109	213.111	9.2	441
GlcN(-Ac)				
GlcN(-Ac)	204.086	204.087	5.9	951
GlcN(-Ac)				

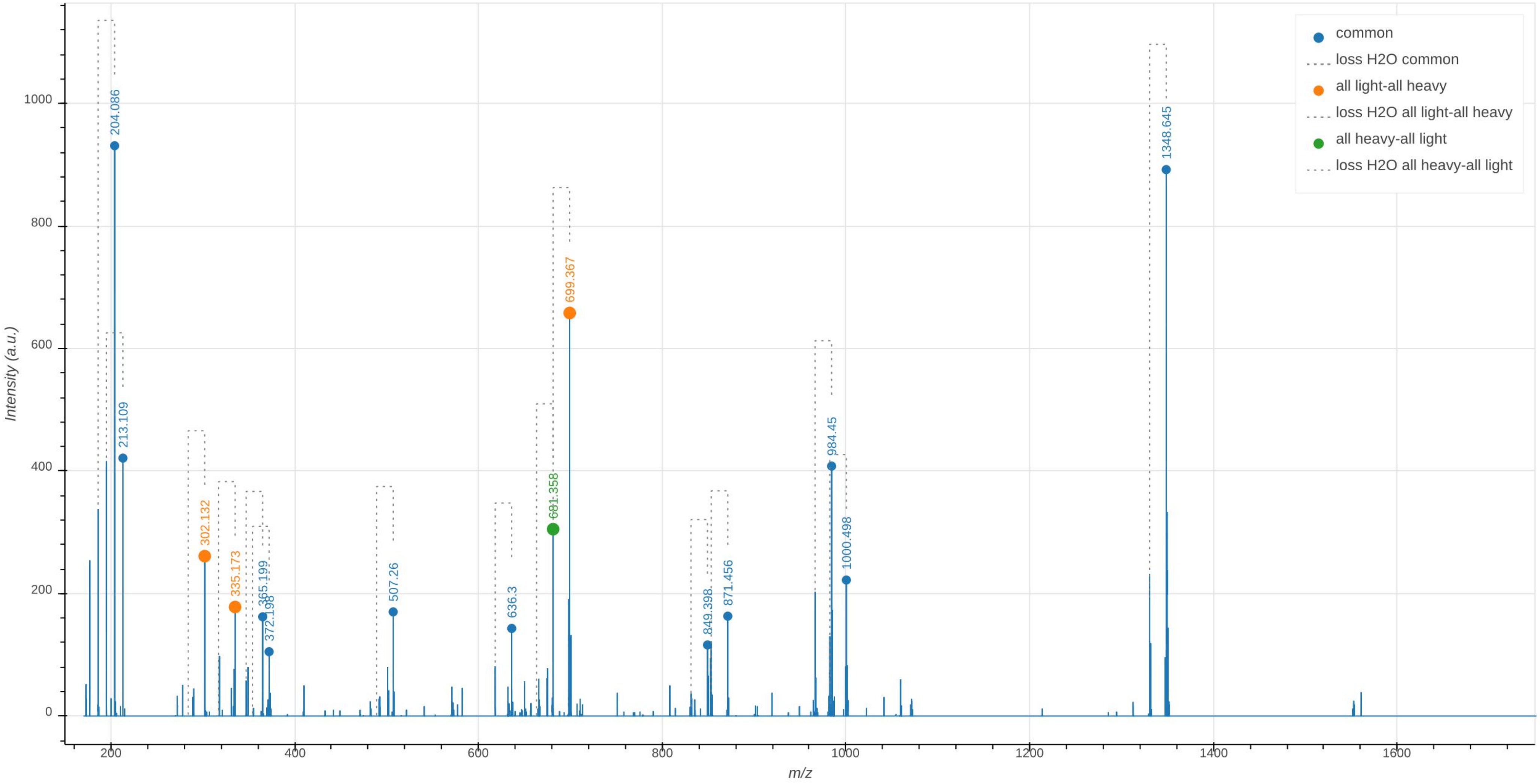
common
loss H2O common
all light-all heavy
loss H2O all light-all heavy
all heavy-all light
loss H2O all heavy-all light
204.086
213.109
302.132
335.173
365.199
372.198
507.26
636.3
681.358
699.367
849.398
871.456
984.45
1000.498
1348.645
Intensity (a.u.)
m/z
0
200
400
600
800
1000
1200
1400
1600

Tetra-Tri

Supplementary Data 4.1: Tandem mass spectrometry analysis of the uniformly labeled Tetra(4→3)Tri dimer. The observed m/z_{obs} and calculated m/z_{cal} values of the parental ion, $[\text{M+2H}]^{2+}$, as determined in the absence of fragmentation (MS1), are indicated. The m/z_{calc} value was used to select the ion for fragmentation. The difference between the m/z_{obs} and m/z_{calc} values is indicated in ppm. The GlcNAc residue is indicated in two moieties, glucosamine (GlcN) and the acetyl group (Ac), since these moieties are potentially differentially labeled. For the same reason, the reduced (Red) MurNAc residue is indicated in three moieties, reduced glucosamine (GlcN^{Red}), the acetyl group (Ac), and the D-lactoyl group. Labeled and unlabeled moieties are represented in red and purple, respectively. The peaks corresponding to the list of fragments appearing in the table are highlighted by orange dots in the mass spectrum. In the mass spectrum, peaks differing by the loss of H_20 are connected by a dashed line. The tables only contain the m/z values for the fragments containing H_20. The arrows indicate the position of the 3→3 and 4→3 crosslinks. Residues in the acceptor are underlined.
An interactive report of the MS2 analysis is available in Supplementary File F4.1.

Precursor ion (MS1)	m/z_{obs} $[\text{M+2H}]^{2+}$	m/z_{calc} $[\text{M+2H}]^{2+}$	ppm
GlcN(-Ac)-GlcN^{Red}(-Ac)-Lac-Ala-Glu-DAP-Ala→DAP-Glu-Ala-Lac-GlcN^{Red}(-Ac)-GlcN(-Ac)	939.992	939.992	0.1

Product ion	m/z_{obs} $[\text{M+H}]^{1+}$	m/z_{calc} $[\text{M+H}]^{1+}$	ppm	Intensity (a.u.)
GlcN(-Ac)-GlcN^{Red}(-Ac)-Lac-Ala-Glu-DAP-Ala→DAP-Glu-Ala-Lac-GlcN^{Red}(-Ac)	1666.880	1666.872	-4.9	113
GlcN^{Red}(-Ac)-Lac-Ala-Glu-DAP-Ala→DAP-Glu-Ala-Lac-GlcN^{Red}(-Ac)	1454.780	1454.769	-7.6	462
GlcN^{Red}(-Ac)-Lac-Ala-Glu-DAP-Ala→DAP-Glu-Ala	1165.627	1165.619	-6.9	103
GlcN^{Red}(-Ac)-Lac-Ala-Glu-DAP-Ala→DAP-Glu	1090.570	1090.574	3.6	240
GlcN^{Red}(-Ac)-Lac-Ala-Glu-DAP-Ala→DAP	955.520	955.518	-2.5	170
Ala-Glu-DAP-Ala→DAP-Glu	801.426	801.424	-2.3	43
Ala→DAP-Glu-Ala-Lac-GlcN^{Red}(-Ac)	774.416	774.416	-0.7	342
GlcN^{Red}(-Ac)-Lac-Ala-Glu-DAP-Ala	756.413	756.405	-11.1	181
Glu-DAP-Ala→DAP-Glu	726.390	726.380	-13.5	50
DAP-Glu-Ala-Lac-GlcN^{Red}(-Ac)	699.369	699.371	2.9	166
GlcN^{Red}(-Ac)-Lac-Ala-Glu-DAP	681.366	681.361	-6.8	54
Ala-Glu-DAP	392.216	392.211	-12.7	44
GlcN(-Ac)	213.112	213.111	-5.5	1238

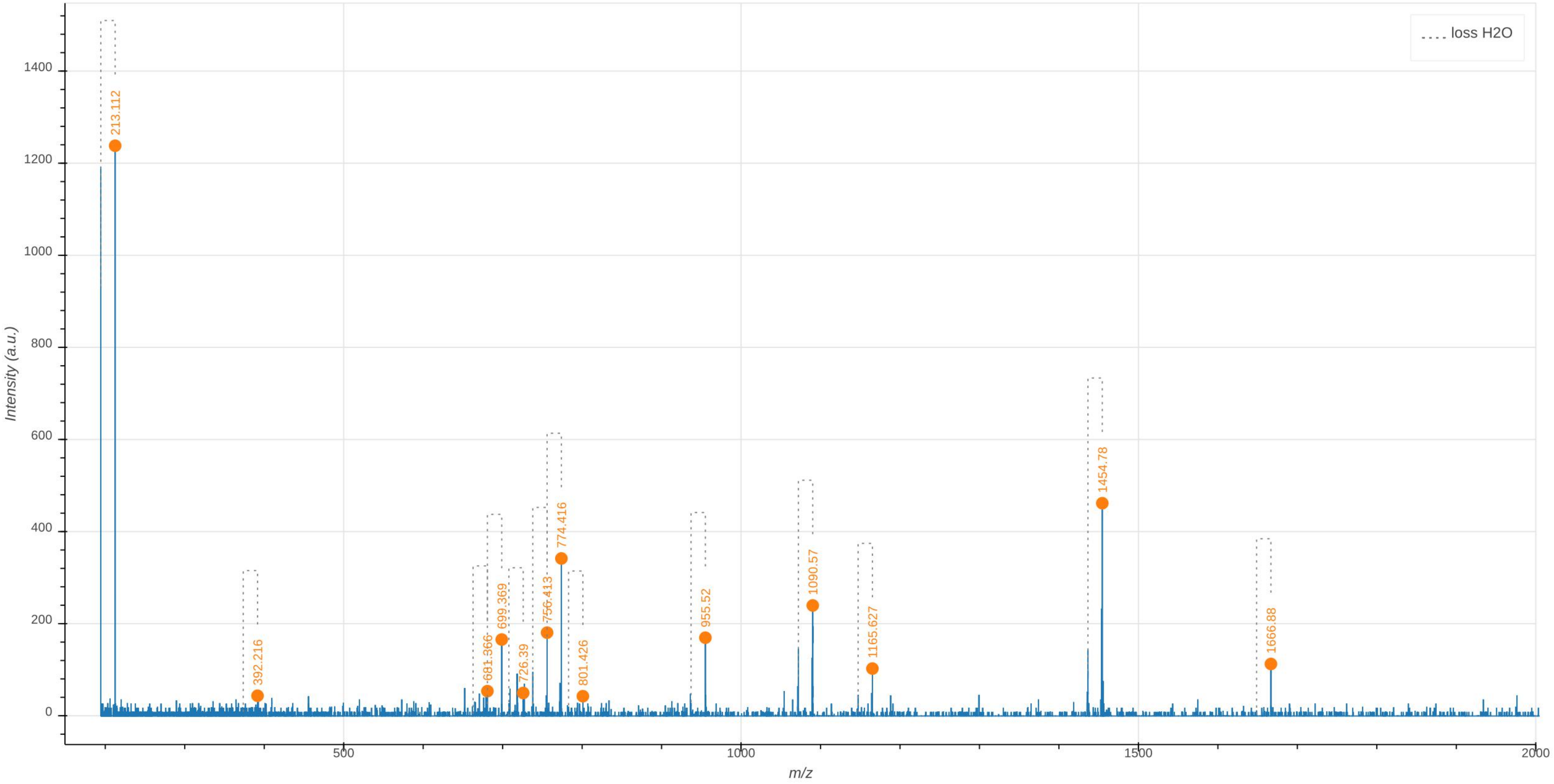

loss H2O
213.112
392.216
681.366
699.369
726.39
756.413
774.416
801.426
955.52
1090.57
1165.627
1454.78
1666.88
Intensity (a.u.)
m/z

Supplementary Data 4.2: Tandem mass spectrometry analysis of the uniformly unlabeled Tri(4→3)Tri dimer. The observed m/z_{obs} and calculated m/z_{cal} values of the parental ion, $[M+2H]^{2+}$, as determined in the absence of fragmentation (MS1), are indicated. The m/z_{calc} value was used to select the ion for fragmentation. The difference between the m/z_{obs} and m/z_{calc} values is indicated in ppm. The GlcNAc residue is indicated in two moieties, glucosamine (GlcN) and the acetyl group (Ac), since these moieties are potentially differentially labeled. For the same reason, the reduced (Red) MurNAc residue is indicated in three moieties, reduced glucosamine ($GlcN^{Red}$), the acetyl group (Ac), and the D-lactoyl group. Labeled and unlabeled moieties are represented in red and purple, respectively. The peaks corresponding to the list of fragments appearing in the table are highlighted by orange dots in the mass spectrum. In the mass spectrum, peaks differing by the loss of H_20 are connected by a dashed line. The tables only contain the m/z values for the fragments containing H_20.
The arrows indicate the position of the 3→3 and 4→3 crosslinks. Residues in the acceptor are underlined.
An interactive report of the MS2 analysis is available in Supplementary File F4.2.

Precursor ion (MS1)	m/z_{obs} [M+2H]$^{2+}$	m/z_{calc} [M+2H]$^{2+}$	ppm
GlcN(-Ac)-GlcN^{Red}(-Ac)-Lac-Ala-Glu-DAP-Ala→DAP-Glu-Ala-Lac-GlcN^{Red}(-Ac)-GlcN(-Ac)	897.889	897.892	-3.3

Product ion	m/z_{obs} [M+H]$^{1+}$	m/z_{calc} [M+H]$^{1+}$	ppm	Intensity (a.u.)
GlcN(-Ac)-GlcN^{Red}(-Ac)-Lac-Ala-Glu-DAP-Ala→DAP-Glu-Ala-Lac-GlcN^{Red}(-Ac)	1591.693	1591.696	1.9	144
GlcN^{Red}(-Ac)-Lac-Ala-Glu-DAP-Ala→DAP-Glu-Ala-Lac-GlcN^{Red}(-Ac)	1388.622	1388.617	-3.4	1802
GlcN^{Red}(-Ac)-Lac-Ala-Glu-DAP-Ala→DAP-Glu-Ala	1111.500	1111.501	0.4	395
GlcN^{Red}(-Ac)-Lac-Ala-Glu-DAP-Ala→DAP-Glu	1040.474	1040.464	-10.3	1464
GlcN^{Red}(-Ac)-Lac-Ala-Glu-DAP-Ala→DAP	911.426	911.421	-5.8	677
Ala-Glu-DAP-Ala→DAP-Glu	763.357	763.347	-13.1	209
Ala→DAP-Glu-Ala-Lac-GlcN^{Red}(-Ac)	739.335	739.336	0.9	2041
GlcN^{Red}(-Ac)-Lac-Ala-Glu-DAP-Ala	721.325	721.326	0.5	1560
Glu-DAP-Ala→DAP-Glu	692.308	692.310	3.2	291
DAP-Glu-Ala-Lac-GlcN^{Red}(-Ac)	668.297	668.299	2.4	618
GlcN^{Red}(-Ac)-Lac-Ala-Glu-DAP	650.288	650.288	1.3	370
Ala-Glu-DAP-Ala→DAP	634.304	634.305	1.7	218
Glu-DAP-Ala→DAP	563.267	563.268	1.2	409
Ala→DAP-Glu-Ala	462.221	462.220	-1.1	137
DAP-Ala→DAP	434.225	434.225	-0.2	298
Ala→DAP-Glu	391.183	391.183	0.3	451
Ala-Glu-DAP	373.173	373.172	-2.5	355
GlcN^{Red}(-Ac)-Lac-Ala	349.161	349.161	-1.1	557
DAP-Glu	320.146	320.146	-1.6	252
Glu-DAP	302.135	302.135	-0.7	366
GlcN^{Red}(-Ac)-Lac	278.125	278.124	-5.0	107

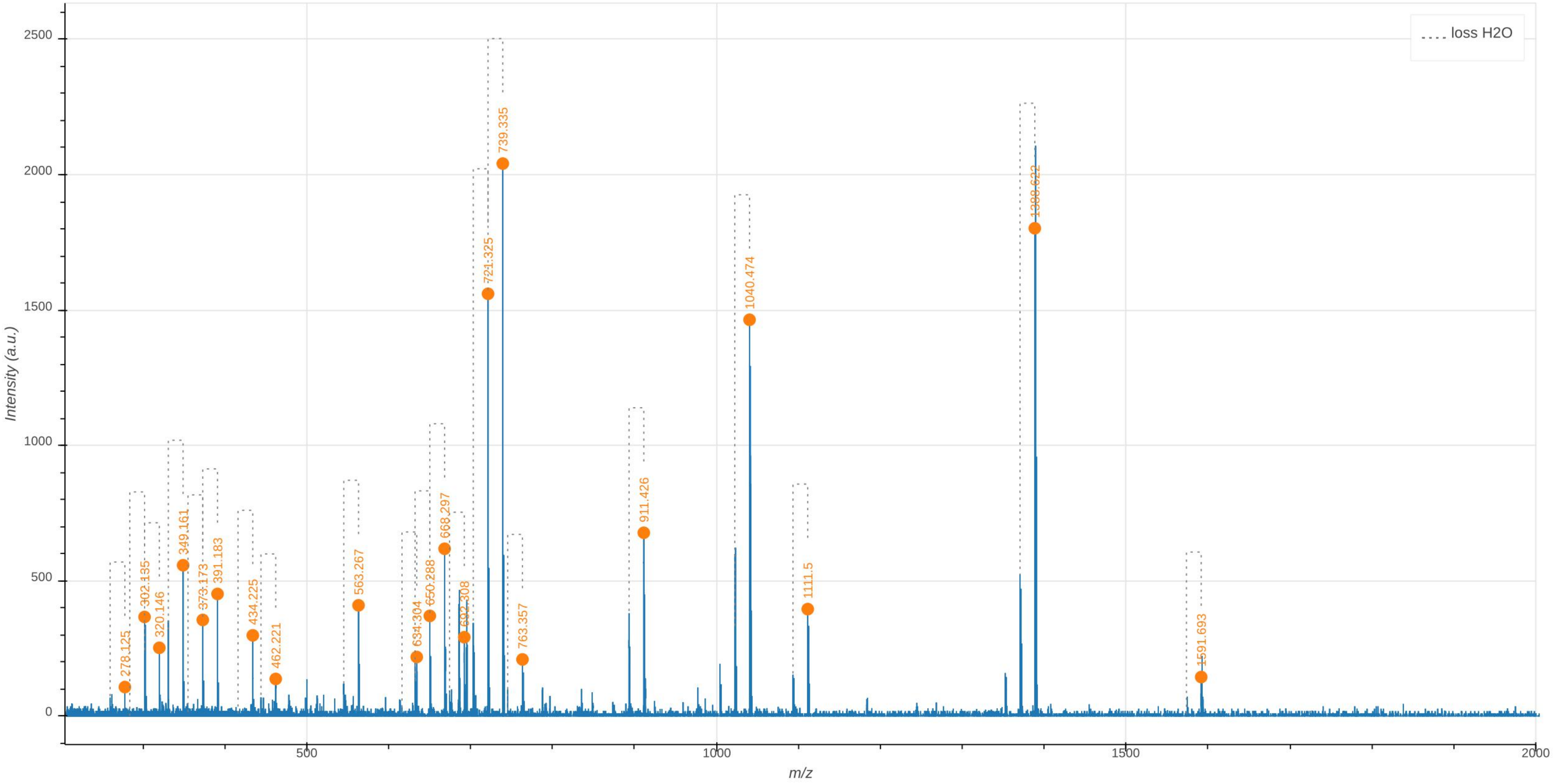

loss H2O
Intensity (a.u.)
m/z
0
500
1000
1500
2000
2500
500
1000
1500
2000
278.125
302.135
320.146
349.161
373.173
391.183
434.225
462.221
563.267
634.304
650.288
668.297
692.308
721.325
739.335
763.357
911.426
1040.474
1111.5
1388.622
1591.693

Supplementary Data 4.3: Tandem mass spectrometry analysis of the Tetra→Tri dimer containing a uniformly labeled disaccharide-tetrapeptide cross-linked to a uniformly unlabeled disaccharide-tripeptide (4→3 cross-link). The observed m/z_{obs} and calculated m/z_{cal} values of the parental ion, $[M+2H]^{2+}$, as determined in the absence of fragmentation (MS1), are indicated. The m/z_{calc} value was used to select the ion for fragmentation. The difference between the m/z_{obs} and m/z_{calc} values is indicated in ppm. The GlcNAc residue is indicated in two moieties, glucosamine (GlcN) and the acetyl group (Ac), since these moieties are potentially differentially labeled. For the same reason, the reduced (Red) MurNAc residue is indicated in three moieties, reduced glucosamine ($\mathrm{GlcN^{Red}}$), the acetyl group (Ac), and the D-lactoyl group. Labeled and unlabeled moieties are represented in red and purple, respectively.

In the mass spectrum, peaks differing by the loss of H_20 are connected by a dashed line. The arrows indicate the position of the 3→3 and 4→3 crosslinks. Residues in the acceptor are underlined.

An interactive report of the MS2 analysis is available in Supplementary File F4.3.

Precursor ion (MS1)	m/z_{obs} $[M+2H]^{2+}$	m/z_{calc} $[M+2H]^{2+}$	ppm
GlcN(-Ac)-GlcN^Red(-Ac)-Lac-Ala-Glu-DAP-Ala→DAP-Glu-Ala-Lac-GlcN^Red(-Ac)-GlcN(-Ac)	917.936	917.940	-4.4

Discriminatory product ion	m/z_{obs} $[M+H]^{1+}$	m/z_{calc} $[M+H]^{1+}$	ppm	Intensity (a.u.)
GlcN^Red(-Ac)-Lac-Ala-Glu-DAP-Ala→DAP-Glu-Ala-Lac-GlcN^Red(-Ac)	1419.689	1419.689	0.3	345
Glu-DAP-Ala→DAP-Glu-Ala-Lac-GlcN^Red(-Ac)	1071.533	1071.536	2.7	71
GlcN^Red(-Ac)-Lac-Ala-Glu-DAP-Ala→DAP-Glu	1055.499	1055.495	-3.4	63
DAP-Ala→DAP-Glu-Ala-Lac-GlcN^Red(-Ac)	942.496	942.493	-3.2	172
GlcN^Red(-Ac)-Lac-Ala-Glu-DAP-Ala→DAP	920.433	920.439	5.9	44
Glu-DAP-Ala→DAP-Glu-Ala	782.389	782.386	-3.8	26
Ala→DAP-Glu-Ala-Lac-GlcN^Red(-Ac)	770.407	770.409	1.4	190
GlcN^Red(-Ac)-Lac-Ala-Glu-DAP-Ala	721.325	721.326	0.7	148
DAP-Glu-Ala-Lac-GlcN^Red(-Ac)	699.381	699.371	-14.3	71
GlcN^Red(-Ac)-Lac-Ala-Glu-DAP	650.294	650.288	-8.5	23
DAP-Ala→DAP-Glu	578.299	578.299	0.2	27
Glu-DAP-Ala→DAP	572.284	572.285	2.7	51
Ala→DAP-Glu-Ala-Lac	556.295	556.290	-9.4	37
Ala-Glu-DAP-Ala	444.207	444.209	6.0	42
DAP-Ala→DAP	443.242	443.243	1.9	81
Ala→DAP-Glu	406.212	406.214	5.0	159
Ala-Glu-DAP	373.173	373.172	-0.5	63
Ala-Lac-GlcN^Red(-Ac)	365.199	365.202	8.0	97
GlcN^Red(-Ac)-Lac-Ala	349.162	349.161	-1.4	40
Glu-DAP	302.134	302.135	4.8	51
GlcN(-Ac)	213.110	213.111	6.5	235
GlcN(-Ac)	204.086	204.087	7.7	502

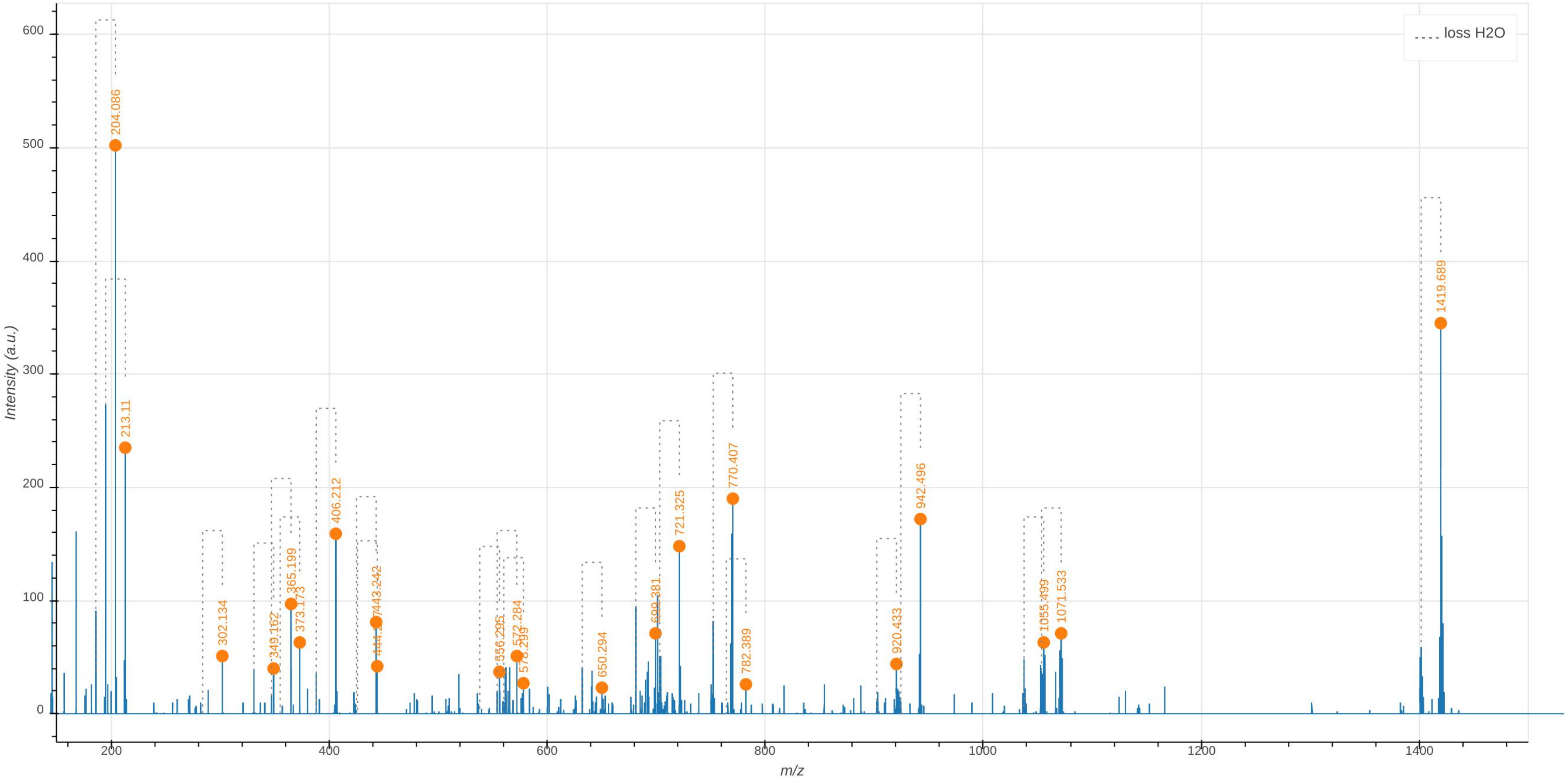

loss H2O
Intensity (a.u.)
m/z
204.086
213.11
302.134
349.162
365.199
373.173
406.212
443.242
556.295
572.284
578.293
650.294
699.381
721.325
770.407
782.389
920.433
942.496
1055.499
1071.533
1419.689

Tri-Tetra

Supplementary Data 5.1: Tandem mass spectrometry analysis of the uniformly labeled Tri(4→3)Tetra dimer. The observed m/z_{obs} and calculated m/z_{cal} values of the parental ion, $[M+2H]^{2+}$, as determined in the absence of fragmentation (MS1), are indicated. The m/z_{calc} value was used to select the ion for fragmentation. The difference between the m/z_{obs} and m/z_{calc} values is indicated in ppm. The GlcNAc residue is indicated in two moieties, glucosamine (GlcN) and the acetyl group (Ac), since these moieties are potentially differentially labeled. For the same reason, the reduced (Red) MurNAc residue is indicated in three moieties, reduced glucosamine (GlcN^{Red}), the acetyl group (Ac), and the D-lactoyl group. Labeled and unlabeled moieties are represented in red and purple, respectively. The peaks corresponding to the list of fragments appearing in the table are highlighted by orange dots in the mass spectrum. In the mass spectrum, peaks differing by the loss of H_20 are connected by a dashed line. The tables only contain the m/z values for the fragments containing H_20. The arrows indicate the position of the 3→3 and 4→3 crosslinks. Residues in the acceptor are underlined.
An interactive report of the MS2 analysis is available in Supplementary File F5.1.

Precursor ion (MS1)	m/z_{obs} $[M+2H]^{2+}$	m/z_{calc} $[M+2H]^{2+}$	ppm
GlcN(-Ac)-GlcN^{Red}(-Ac)-Lac-Ala-Glu-DAP→DAP(-Ala)-Glu-Ala-Lac-GlcN^{Red}(-Ac)-GlcN(-Ac)	939.988	939.992	-3.3

Product ion	m/z_{obs} $[M+H]^{1+}$	m/z_{calc} $[M+H]^{1+}$	ppm	Intensity (a.u.)
GlcN^{Red}(-Ac)-Lac-Ala-Glu-DAP→DAP(-Ala)-Glu-Ala-Lac-GlcN^{Red}(-Ac)	1454.757	1454.769	8.0	376
GlcN^{Red}(-Ac)-Lac-Ala-Glu-DAP→DAP(-Ala)-Glu-Ala	1165.632	1165.619	-11.3	41
GlcN^{Red}(-Ac)-Lac-Ala-Glu-DAP→DAP(-Ala)-Glu	1090.586	1090.574	-10.9	238
GlcN^{Red}(-Ac)-Lac-Ala-Glu-DAP→DAP-Glu-Ala	1072.561	1072.564	2.6	158
GlcN^{Red}(-Ac)-Lac-Ala-Glu-DAP→DAP-Glu	997.520	997.520	-0.5	97
GlcN^{Red}(-Ac)-Lac-Ala-Glu-DAP→DAP(-Ala)	955.528	955.518	-10.1	135
DAP(-Ala)-Glu-Ala-Lac-GlcN^{Red}(-Ac)	774.414	774.416	2.5	184
Glu-DAP→DAP(-Ala)	591.320	591.324	6.3	44
GlcN^{Red}(-Ac)-Lac-Ala	365.201	365.202	1.9	135
Glu-DAP	317.166	317.167	3.2	123

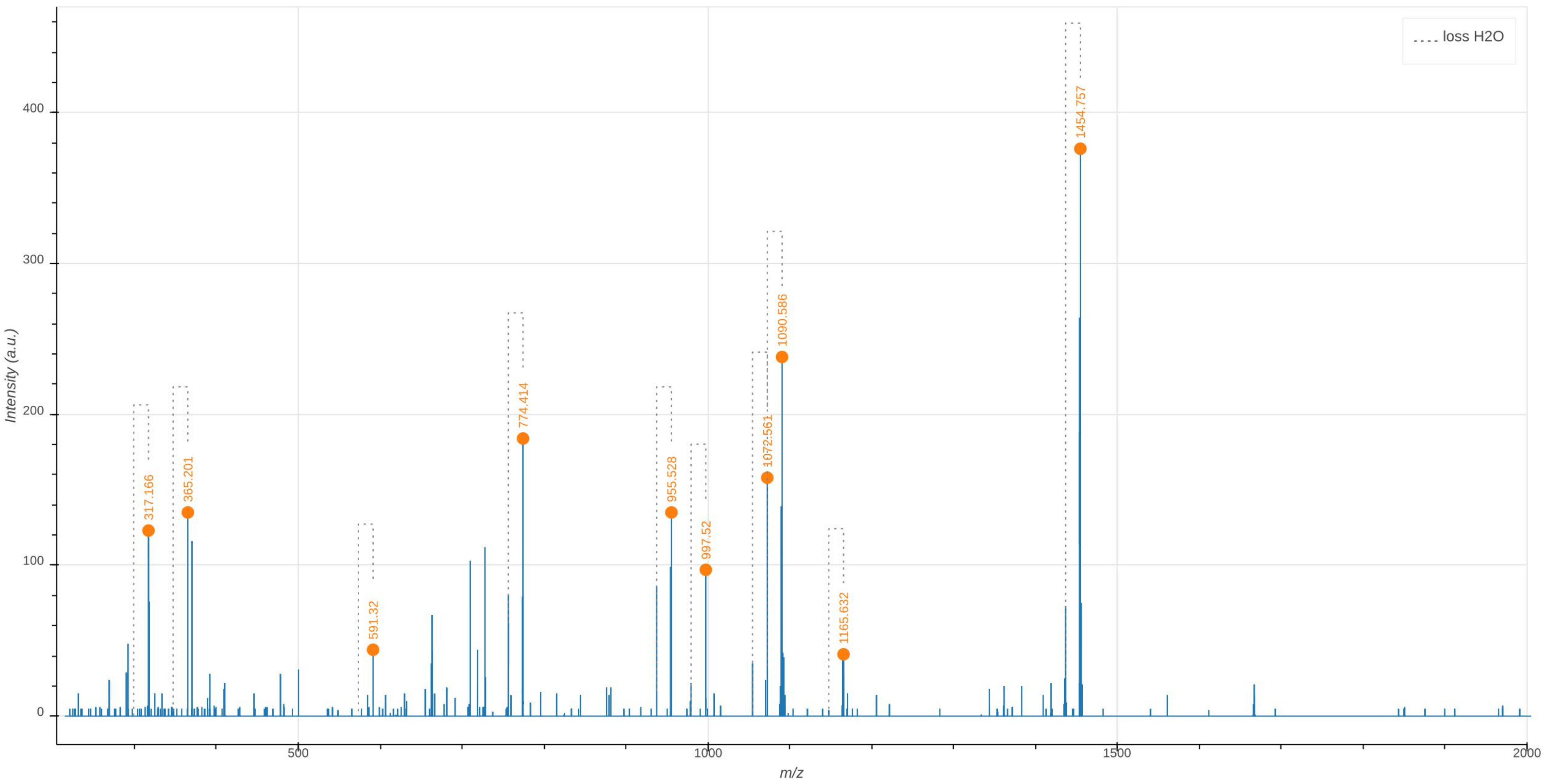

loss H2O
317.166
365.201
591.32
774.414
955.528
997.52
1072.561
1090.586
1165.632
1454.757
Intensity (a.u.)
0
100
200
300
400
500
1000
1500
2000
m/z

Supplementary Data 5.2: Tandem mass spectrometry analysis of the uniformly unlabeled Tri(3→3)Tetra dimer. The observed m/z_{obs} and calculated m/z_{cal} values of the parental ion, $[\text{M+2H}]^{2+}$, as determined in the absence of fragmentation (MS1), are indicated. The m/z_{calc} value was used to select the ion for fragmentation. The difference between the m/z_{obs} and m/z_{calc} values is indicated in ppm. The GlcNAc residue is indicated in two moieties, glucosamine (GlcN) and the acetyl group (Ac), since these moieties are potentially differentially labeled. For the same reason, the reduced (Red) MurNAc residue is indicated in three moieties, reduced glucosamine (GlcN^{Red}), the acetyl group (Ac), and the D-lactoyl group. Labeled and unlabeled moieties are represented in red and purple, respectively. The peaks corresponding to the list of fragments appearing in the table are highlighted by orange dots in the mass spectrum. In the mass spectrum, peaks differing by the loss of H_20 are connected by a dashed line. The tables only contain the m/z values for the fragments containing H_20.
The arrows indicate the position of the 3→3 and 4→3 crosslinks. Residues in the acceptor are underlined.
An interactive report of the MS2 analysis is available in Supplementary File F5.2.

Precursor ion (MS1)	m/z_{obs} [M+2H]$^{2+}$	m/z_{calc} [M+2H]$^{2+}$	ppm
GlcN(-Ac)-GlcN^{Red}(-Ac)-Lac-Ala-Glu-DAP→DAP(-Ala)-Glu-Ala-Lac-GlcN^{Red}(-Ac)-GlcN(-Ac)	897.891	897.892	-0.9

Product ion	m/z_{obs} [M+H]$^{1+}$	m/z_{calc} [M+H]$^{1+}$	ppm	Intensity (a.u.)
GlcN(-Ac)-GlcN^{Red}(-Ac)-Lac-Ala-Glu-DAP→DAP(-Ala)-Glu-Ala-Lac-GlcN^{Red}(-Ac)	1591.703	1591.696	-4.5	290
GlcN^{Red}(-Ac)-Lac-Ala-Glu-DAP→DAP(-Ala)-Glu-Ala-Lac-GlcN^{Red}(-Ac)	1388.618	1388.617	-0.8	2966
GlcN^{Red}(-Ac)-Lac-Ala-Glu-DAP→DAP-Glu-Ala-Lac-GlcN^{Red}(-Ac)	1299.575	1299.569	-4.2	311
GlcN^{Red}(-Ac)-Lac-Ala-Glu-DAP→DAP(-Ala)-Glu-Ala-Lac	1183.529	1183.522	-5.7	139
GlcN^{Red}(-Ac)-Lac-Ala-Glu-DAP→DAP(-Ala)-Glu-Ala	1111.500	1111.501	0.4	614
GlcN^{Red}(-Ac)-Lac-Ala-Glu-DAP→DAP-Glu-Ala-Lac	1094.502	1094.474	-25.7	132
GlcN^{Red}(-Ac)-Lac-Ala-Glu-DAP→DAP(-Ala)-Glu	1040.465	1040.464	-1.8	1697
GlcN^{Red}(-Ac)-Lac-Ala-Glu-DAP→DAP-Glu-Ala	1022.450	1022.453	2.8	687
GlcN^{Red}(-Ac)-Lac-Ala-Glu-DAP→DAP-Glu	951.412	951.416	4.5	820
GlcN^{Red}(-Ac)-Lac-Ala-Glu-DAP→DAP(-Ala)	911.422	911.421	-0.9	805
Lac-Ala-Glu-DAP→DAP(-Ala)-Glu-Ala	906.399	906.406	7.8	112
GlcN^{Red}(-Ac)-Lac-Ala-Glu-DAP→DAP	822.372	822.373	1.4	141
Ala-Glu-DAP→DAP(-Ala)-Glu	763.350	763.347	-3.4	207
DAP(-Ala)-Glu-Ala-Lac-GlcN^{Red}(-Ac)	739.335	739.336	0.9	1449
Glu-DAP→DAP(-Ala)-Glu	692.308	692.310	3.2	351
Ala-Glu-DAP→DAP-Glu	674.295	674.300	6.8	209
GlcN^{Red}(-Ac)-Lac-Ala-Glu-DAP	650.291	650.288	-3.1	639
Ala-Glu-DAP→DAP(-Ala)	634.304	634.305	1.8	231
Glu-DAP→DAP-Glu	603.267	603.263	-6.7	231
Glu-DAP→DAP(-Ala)	563.270	563.268	-4.0	605
Ala-Glu-DAP→DAP	545.260	545.257	-4.6	275
GlcN^{Red}(-Ac)-Lac-Ala-Glu	478.201	478.204	5.9	120
Glu-DAP→DAP	474.220	474.220	-1.0	198
DAP→DAP(-Ala)	434.225	434.225	-0.2	182
DAP(-Ala)-Glu	391.180	391.183	7.9	360
Ala-Glu-DAP	373.173	373.172	-2.4	360
GlcN^{Red}(-Ac)-Lac-Ala	349.160	349.161	3.2	653
Glu-DAP	302.135	302.135	-0.7	1087
GlcN^{Red}(-Ac)-Lac	278.124	278.124	0.3	167

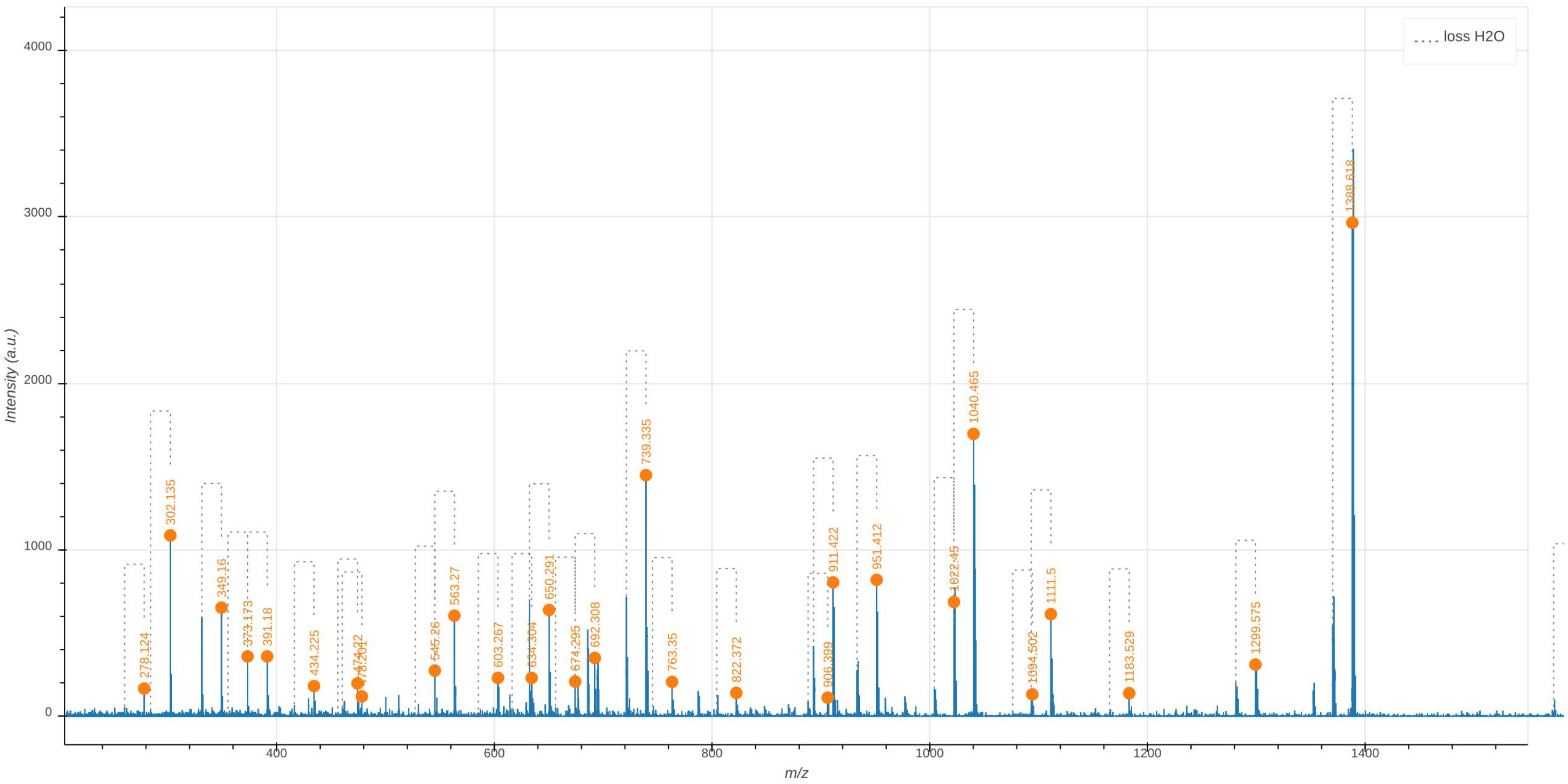

loss H2O
Intensity (a.u.)
m/z
4000
3000
2000
1000
0
400
600
800
1000
1200
1400
278.124
302.135
349.16
373.173
391.18
434.225
474.22
478.201
545.26
563.27
603.267
634.304
650.291
674.295
692.308
739.335
763.35
822.372
906.399
911.422
951.412
1022.45
1040.465
1094.502
1111.5
1183.529
1299.575
1388.618

Supplementary Data 5.3: Tandem mass spectrometry analysis of the Tri→Tetra dimer containing a uniformly labeled disaccharide-tetrapeptide cross-linked to a uniformly unlabeled disaccharide-tripeptide (3→3 cross-link). The observed m/z_{obs} and calculated m/z_{cal} values of the parental ion, $[\mathrm{M+2H}]^{2+}$, as determined in the absence of fragmentation (MS1), are indicated. The m/z_{calc} value was used to select the ion for fragmentation. The difference between the m/z_{obs} and m/z_{calc} values is indicated in ppm. The GlcNAc residue is indicated in two moieties, glucosamine (GlcN) and the acetyl group (Ac), since these moieties are potentially differentially labeled. For the same reason, the reduced (Red) MurNAc residue is indicated in three moieties, reduced glucosamine ($\mathrm{GlcN}^{\mathrm{Red}}$), the acetyl group (Ac), and the D-lactoyl group. Labeled and unlabeled moieties are represented in red and purple, respectively.

In the mass spectrum, peaks differing by the loss of H_20 are connected by a dashed line. The arrows indicate the position of the 3→3 and 4→3 crosslinks. Residues in the acceptor are underlined.

An interactive report of the MS2 analysis is available in Supplementary File F5.3.

Precursor ion (MS1)	m/z_{obs} [M+2H]$^{2+}$	m/z_{calc} [M+2H]$^{2+}$	ppm
GlcN(-Ac)-GlcN^{Red}(-Ac)-Lac-Ala-Glu-DAP→DAP(-Ala)-Glu-Ala-Lac-GlcN^{Red}(-Ac)-GlcN(-Ac)	919.942	919.943	-1.0

Product ion	m/z_{obs} [M+H]$^{1+}$	m/z_{calc} [M+H]$^{1+}$	ppm	Intensity (a.u.)
GlcN^{Red}(-Ac)-Lac-Ala-Glu-DAP→DAP(-Ala)-Glu-Ala-Lac-GlcN^{Red}(-Ac)	1423.693	1423.696	2.4	167
GlcN^{Red}(-Ac)-Lac-Ala-Glu-DAP→DAP-Glu-Ala-Lac-GlcN^{Red}(-Ac)	1330.655	1330.642	-10.1	30
Glu-DAP→DAP(-Ala)-Glu-Ala-Lac-GlcN^{Red}(-Ac)	1075.541	1075.543	2.3	41
GlcN^{Red}(-Ac)-Lac-Ala-Glu-DAP→DAP(-Ala)-Glu	1059.504	1059.502	-1.7	58
Glu-DAP→DAP-Glu-Ala-Lac-GlcN^{Red}(-Ac)	982.486	982.488	2.8	13
DAP→DAP(-Ala)-Glu-Ala-Lac-GlcN^{Red}(-Ac)	946.496	946.500	5.1	37
GlcN^{Red}(-Ac)-Lac-Ala-Glu-DAP→DAP(-Ala)	924.445	924.446	1.1	57
DAP(-Ala)-Glu-Ala-Lac-GlcN^{Red}(-Ac)	774.421	774.416	-7.2	105
Glu-Ala-Lac-GlcN^{Red}(-Ac)-GlcN(-Ac)	712.348	712.362	19.6	17
DAP-Glu-Ala-Lac-GlcN^{Red}(-Ac)	681.354	681.361	10.6	19
Glu-DAP→DAP-Glu	618.291	618.294	4.1	29
DAP→DAP-Glu	489.249	489.251	5.0	15
DAP→DAP(-Ala)	447.248	447.250	4.1	15
DAP-Glu-Ala	392.206	392.211	13.2	40
GlcN^{Red}(-Ac)-Lac-Ala	349.157	349.161	10.7	18
DAP-Glu	317.170	317.167	-9.7	15
GlcN(-Ac)	213.110	213.111	5.5	122
GlcN(-Ac)	204.086	204.087	6.8	471
DAP	182.110	182.110	0.8	24
Ala-Lac	151.083	151.083	-1.5	40

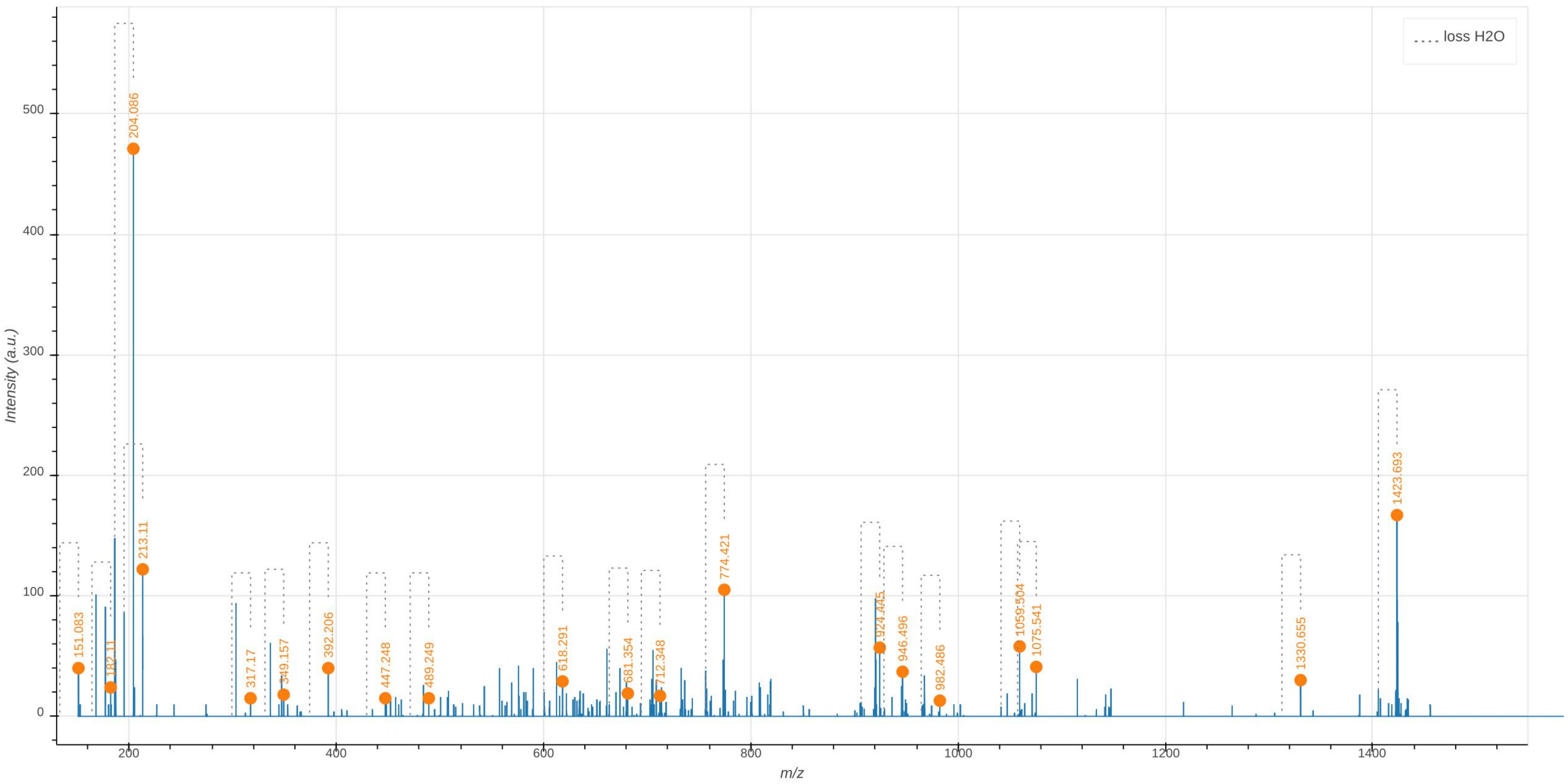

loss H2O
Intensity (a.u.)
m/z
204.086
213.11
151.083
182.11
317.17
349.157
392.206
447.248
489.249
618.291
681.354
712.348
774.421
924.445
946.496
982.486
1059.504
1075.541
1330.655
1423.693

Tetra-Tetra

Supplementary Data 6.1: Tandem mass spectrometry analysis of the uniformly labeled Tetra(4→3)Tetra dimer. The observed m/z_{obs} and calculated m/z_{cal} values of the parental ion, $[M+2H]^{2+}$, as determined in the absence of fragmentation (MS1), are indicated. The m/z_{calc} value was used to select the ion for fragmentation. The difference between the m/z_{obs} and m/z_{calc} values is indicated in ppm. The GlcNAc residue is indicated in two moieties, glucosamine (GlcN) and the acetyl group (Ac), since these moieties are potentially differentially labeled. For the same reason, the reduced (Red) MurNAc residue is indicated in three moieties, reduced glucosamine (GlcN^{Red}), the acetyl group (Ac), and the D-lactoyl group. Labeled and unlabeled moieties are represented in red and purple, respectively. The peaks corresponding to the list of fragments appearing in the table are highlighted by orange dots in the mass spectrum. In the mass spectrum, peaks differing by the loss of H_20 are connected by a dashed line. The tables only contain the m/z values for the fragments containing H_20. The arrows indicate the position of the 3→3 and 4→3 crosslinks. Residues in the acceptor are underlined.
An interactive report of the MS2 analysis is available in Supplementary File F6.1.

Precursor ion (MS1)	m/z_{obs} $[M+2H]^{2+}$	m/z_{calc} $[M+2H]^{2+}$	ppm
GlcN(-Ac)-GlcN^{Red}(-Ac)-Lac-Ala-Glu-DAP-Ala→DAP(-Ala)-Glu-Ala-Lac-GlcN^{Red}(-Ac)-GlcN(-Ac)	977.512	977.514	-2.0

Product ion	m/z_{obs} $[M+H]^{1+}$	m/z_{calc} $[M+H]^{1+}$	ppm	Intensity (a.u.)
GlcN^{Red}(-Ac)-Lac-Ala-Glu-DAP-Ala→DAP(-Ala)-Glu-Ala-Lac-GlcN^{Red}(-Ac)	1529.815	1529.813	-1.7	1017
GlcN^{Red}(-Ac)-Lac-Ala-Glu-DAP-Ala→DAP(-Ala)-Glu-Ala	1240.686	1240.663	-18.5	172
GlcN^{Red}(-Ac)-Lac-Ala-Glu-DAP-Ala→DAP(-Ala)-Glu	1165.619	1165.619	-0.4	576
GlcN^{Red}(-Ac)-Lac-Ala-Glu-DAP-Ala→DAP-Glu-Ala	1147.614	1147.608	-5.1	229
GlcN^{Red}(-Ac)-Lac-Ala-Glu-DAP-Ala→DAP-Glu	1072.569	1072.564	-4.8	156
GlcN^{Red}(-Ac)-Lac-Ala-Glu-DAP-Ala→DAP(-Ala)	1030.565	1030.562	-2.9	194
Ala→DAP(-Ala)-Glu-Ala-Lac-GlcN^{Red}(-Ac)	849.462	849.460	-2.4	405
DAP(-Ala)-Glu-Ala-Lac-GlcN^{Red}(-Ac)	774.421	774.416	-7.4	274
GlcN^{Red}(-Ac)-Lac-Ala-Glu-DAP-Ala	756.411	756.405	-8.2	533
Glu-DAP-Ala→DAP-Glu	708.373	708.370	-5.4	106
GlcN^{Red}(-Ac)-Lac-Ala	365.203	365.202	-1.1	320
Glu-DAP	317.168	317.167	-4.8	197
GlcN(-Ac)	213.112	213.111	-6.5	2182
DAP	182.113	182.110	-14.4	253
Lac-Ala	151.086	151.083	-19.2	414

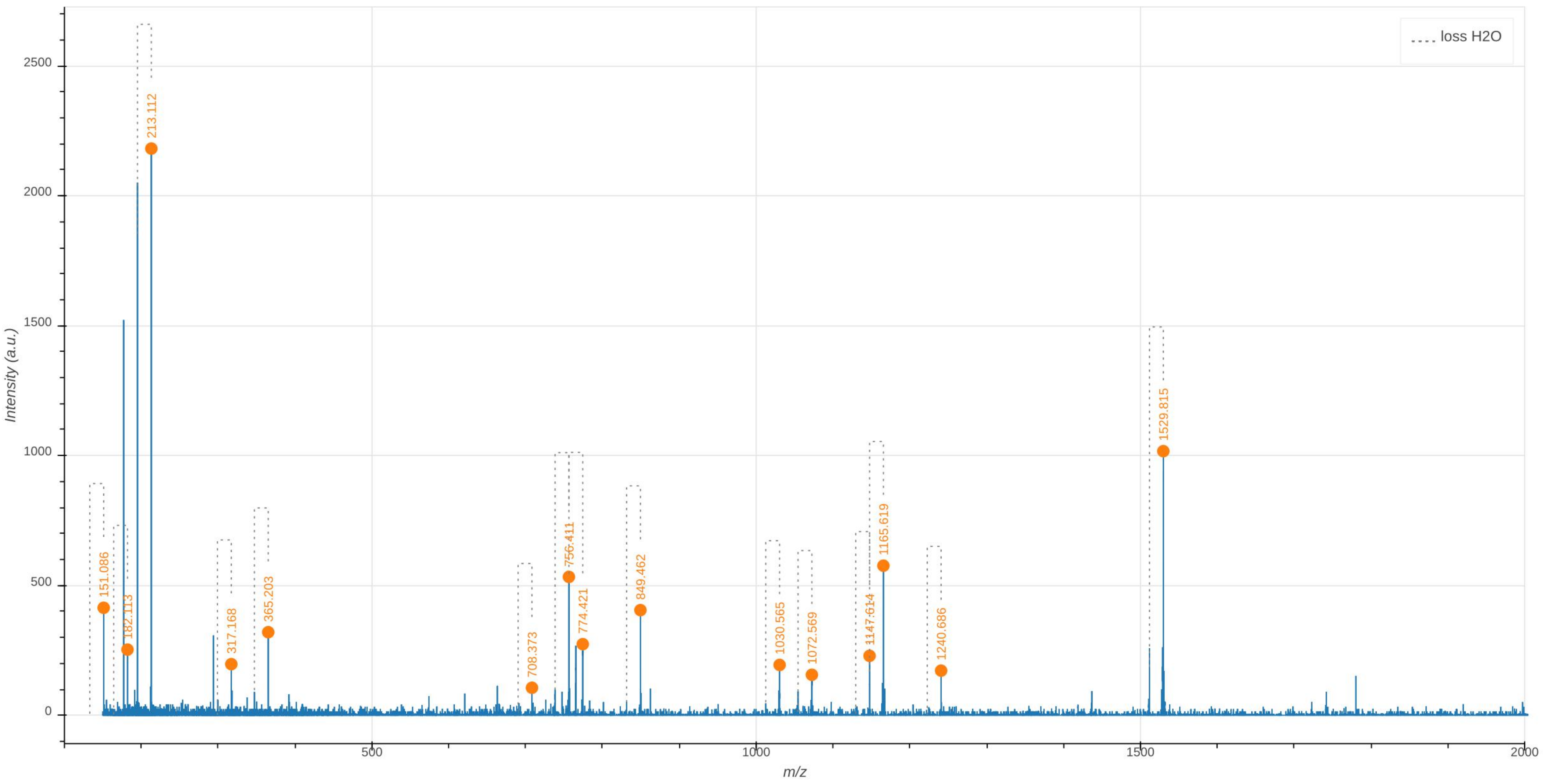

loss H2O
Intensity (a.u.)
m/z
0
500
1000
1500
2000
2500
151.086
182.113
213.112
317.168
365.203
708.373
756.411
774.421
849.462
1030.565
1072.569
1147.614
1165.619
1240.686
1529.815

Supplementary Data 6.2: Tandem mass spectrometry analysis of the uniformly unlabeled Tetra(4→3)Tetra dimer. The observed m/z_{obs} and calculated m/z_{cal} values of the parental ion, $[M+2H]^{2+}$, as determined in the absence of fragmentation (MS1), are indicated. The m/z_{calc} value was used to select the ion for fragmentation. The difference between the m/z_{obs} and m/z_{calc} values is indicated in ppm. The GlcNAc residue is indicated in two moieties, glucosamine (GlcN) and the acetyl group (Ac), since these moieties are potentially differentially labeled. For the same reason, the reduced (Red) MurNAc residue is indicated in three moieties, reduced glucosamine (GlcN^{Red}), the acetyl group (Ac), and the D-lactoyl group. Labeled and unlabeled moieties are represented in red and purple, respectively. The peaks corresponding to the list of fragments appearing in the table are highlighted by orange dots in the mass spectrum. In the mass spectrum, peaks differing by the loss of H_20 are connected by a dashed line. The tables only contain the m/z values for the fragments containing H_20.
The arrows indicate the position of the 3→3 and 4→3 crosslinks. Residues in the acceptor are underlined.
An interactive report of the MS2 analysis is available in Supplementary File F6.2.

Precursor ion (MS1)	m/z_{obs} $[M+2H]^{2+}$	m/z_{calc} $[M+2H]^{2+}$	ppm
GlcN(-Ac)-GlcN^{Red}(-Ac)-Lac-Ala-Glu-DAP-Ala→DAP(-Ala)-Glu-Ala-Lac-GlcN^{Red}(-Ac)-GlcN(-Ac)	933.409	933.410	-1.7

Product ion	m/z_{obs} $[M+H]^{1+}$	m/z_{calc} $[M+H]^{1+}$	ppm	Intensity (a.u.)
GlcN(-Ac)-GlcN^{Red}(-Ac)-Lac-Ala-Glu-DAP-Ala→DAP(-Ala)-Glu-Ala-Lac-GlcN^{Red}(-Ac)	1662.731	1662.733	1.1	416
GlcN^{Red}(-Ac)-Lac-Ala-Glu-DAP-Ala→DAP(-Ala)-Glu-Ala-Lac-GlcN^{Red}(-Ac)	1459.655	1459.654	-0.9	4041
GlcN^{Red}(-Ac)-Lac-Ala-Glu-DAP-Ala→DAP-Glu-Ala-Lac-GlcN^{Red}(-Ac)	1370.614	1370.606	-5.8	419
GlcN(-Ac)-GlcN^{Red}(-Ac)-Lac-Ala-Glu-DAP-Ala→DAP(-Ala)-Glu	1314.586	1314.580	-4.8	108
GlcN^{Red}(-Ac)-Lac-Ala-Glu-DAP-Ala→DAP(-Ala)-Glu-Ala	1182.533	1182.538	4.2	436
GlcN^{Red}(-Ac)-Lac-Ala-Glu-DAP-Ala→DAP(-Ala)-Glu	1111.500	1111.501	0.3	2489
GlcN^{Red}(-Ac)-Lac-Ala-Glu-DAP-Ala→DAP-Glu-Ala	1093.492	1093.490	-1.8	1176
GlcN^{Red}(-Ac)-Lac-Ala-Glu-DAP-Ala→DAP-Glu	1022.450	1022.453	3.2	1094
GlcN^{Red}(-Ac)-Lac-Ala-Glu-DAP-Ala→DAP(-Ala)	982.461	982.458	-2.7	1305
GlcN^{Red}(-Ac)-Lac-Ala-Glu-DAP-Ala→DAP	893.408	893.410	3.2	159
Ala-Glu-DAP-Ala→DAP(-Ala)-Glu	834.397	834.385	-14.7	173
Ala→DAP(-Ala)-Glu-Ala-Lac-GlcN^{Red}(-Ac)	810.373	810.373	0.4	2603
Glu-DAP-Ala→DAP(-Ala)-Glu	763.353	763.347	-7.3	355
Ala-Glu-DAP-Ala→DAP-Glu	745.337	745.337	0.4	285
DAP(-Ala)-Glu-Ala-Lac-GlcN^{Red}(-Ac)	739.336	739.336	0.8	1027
GlcN^{Red}(-Ac)-Lac-Ala-Glu-DAP-Ala	721.328	721.326	-3.7	2676
Ala-Glu-DAP-Ala→DAP(-Ala)	705.345	705.342	-3.8	134
Glu-DAP-Ala→DAP-Glu	674.300	674.300	0.1	133
GlcN^{Red}(-Ac)-Lac-Ala-Glu-DAP	650.291	650.288	-3.3	234
Glu-DAP-Ala→DAP(-Ala)	634.304	634.305	1.6	324
Ala-Glu-DAP-Ala→DAP	616.294	616.294	-0.2	193
Glu-DAP-Ala→DAP	545.257	545.257	0.6	279
Ala→DAP(-Ala)-Glu-Ala	533.257	533.257	0.6	121
DAP-Ala→DAP(-Ala)	505.261	505.262	2.2	228
Ala→DAP(-Ala)-Glu	462.221	462.220	-1.2	549
Ala-Glu-DAP-Ala	444.209	444.209	0.1	198
DAP(-Ala)-Glu	391.183	391.183	0.2	328
Ala-Glu-DAP	373.173	373.172	-2.6	787
GlcN^{Red}(-Ac)-Lac-Ala	349.162	349.161	-1.2	1062
Ala→DAP(-Ala)	333.177	333.177	2.6	284
Glu-DAP	302.135	302.135	-0.9	560
GlcN^{Red}(-Ac)-Lac	278.121	278.124	10.8	141

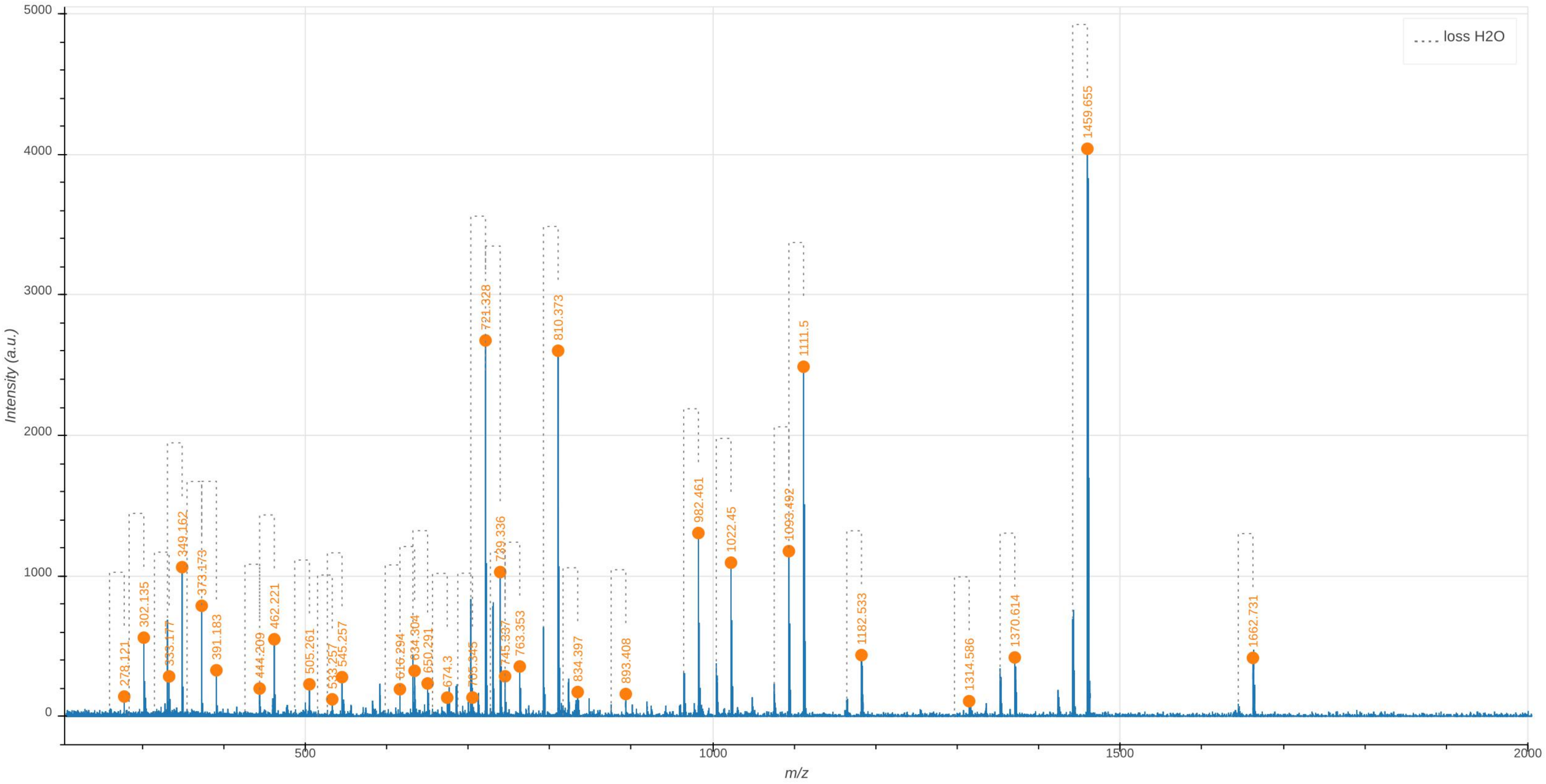
loss H2O
Intensity (a.u.)
m/z
0
1000
2000
3000
4000
5000
500
1000
1500
2000
278.121
302.135
333.177
349.162
373.173
391.183
444.209
462.221
505.261
533.257
545.257
616.294
634.304
650.291
674.3
705.343
721.328
739.336
745.337
763.353
810.373
834.397
893.408
982.461
1022.45
1093.492
1111.5
1182.533
1314.586
1370.614
1459.655
1662.731

Supplementary Data 6.3: Tandem mass spectrometry analysis of the Tetra→Tetra dimer containing a uniformly labeled disaccharide-tripeptide cross-linked to a uniformly unlabeled disaccharide-tripeptide (4→3 cross-link). The observed $m/z_{\rm obs}$ and calculated $m/z_{\rm cal}$ values of the parental ion, $[M+2H]^{2+}$, as determined in the absence of fragmentation (MS1), are indicated. The $m/z_{\rm calc}$ value was used to select the ion for fragmentation. The difference between the $m/z_{\rm obs}$ and $m/z_{\rm calc}$ values is indicated in ppm. The GlcNAc residue is indicated in two moieties, glucosamine (GlcN) and the acetyl group (Ac), since these moieties are potentially differentially labeled. For the same reason, the reduced (Red) MurNAc residue is indicated in three moieties, reduced glucosamine ($\mathrm{GlcN^{Red}}$), the acetyl group (Ac), and the D-lactoyl group. Labeled and unlabeled moieties are represented in red and purple, respectively. The fragmentation potentially leads to two series of fragments (discriminatory fragments) specific of the isotopomers containing the fully labeled disaccharide subunit in the donor (all heavy-all light) or acceptor position (all light-all heavy). These fragments are highlighted by green and orange dots in the mass spectrum, respectively. The other fragments are common to the fragmentation of the two isotopomers (highlighted by blue dots in the mass spectrum). The mass spectrum indicates the presence of the all light-all heavy isotopomers (orange dots). The peak at m/z_{obs} 1128.568 (green dot) can also be accounted for by the loss of H_20 from the peak at 1142.567 indicating that all peaks may be accounted for by the presence of the all light-all heavy isotopomer.

In the mass spectrum, peaks differing by the loss of H_20 are connected by a dashed line. The arrows indicate the position of the 3→3 and 4→3 crosslinks. Residues in the acceptor are underlined.

An interactive report of the MS2 analysis is available in Supplementary File F6.3.

Precursor ion (MS1)	m/z_{obs} [M+2H]$^{2+}$	m/z_{calc} [M+2H]$^{2+}$	ppm
GlcN(-Ac)-GlcN^{Red}(-Ac)-Lac-Ala-Glu-DAP-Ala→DAP(-Ala)-Glu-Ala-Lac-GlcN^{Red}(-Ac)-GlcN(-Ac)	955.459	955.462	-2.6
GlcN(-Ac)-GlcN^{Red}(-Ac)-Lac-Ala-Glu-DAP-Ala→DAP(-Ala)-Glu-Ala-Lac-GlcN^{Red}(-Ac)-GlcN(-Ac)	955.459	955.462	-2.6

Discriminatory product ion	m/z_{obs} [M+H]$^{1+}$	m/z_{calc} [M+H]$^{1+}$	ppm	Intensity (a.u.)	Isotopologue
GlcN^{Red}(-Ac)-Lac-Ala-Glu-DAP-Ala→DAP-Glu-Ala	1128.568	1128.570	1.6	215	all heavy-all light
Glu-DAP-Ala→DAP-Glu-Ala-Lac-GlcN^{Red}(-Ac)	1053.516	1053.525	9.2	97	all light-all heavy
GlcN^{Red}(-Ac)-Lac-Ala-Glu-DAP-Ala→DAP-Glu	1037.486	1037.484	-1.5	153	all light-all heavy
Ala→DAP(-Ala)-Glu-Ala-Lac-GlcN^{Red}(-Ac)	845.449	845.453	4.3	304	all light-all heavy
DAP(-Ala)-Glu-Ala-Lac-GlcN^{Red}(-Ac)	774.410	774.416	7.5	459	all light-all heavy
Ala→DAP-Glu-Ala-Lac-GlcN^{Red}(-Ac)	752.394	752.398	5.1	307	all light-all heavy
GlcN^{Red}(-Ac)-Lac-Ala-Glu-DAP-Ala	721.322	721.326	4.4	186	all light-all heavy
Glu-DAP-Ala→DAP-Glu	689.334	689.331	-3.8	139	all light-all heavy
DAP(-Ala)-Glu	410.225	410.221	-9.5	158	all light-all heavy
Ala→DAP-Glu	388.202	388.204	4.3	128	all light-all heavy
Ala→DAP(-Ala)	346.197	346.202	14.6	106	all light-all heavy

Common product ion	m/z_{obs} [M+H]$^{1+}$	m/z_{calc} [M+H]$^{1+}$	ppm	Intensity (a.u.)
GlcN^{Red}(-Ac)-Lac-Ala-Glu-DAP-Ala→DAP(-Ala)-Glu-Ala-Lac-GlcN^{Red}(-Ac)	1494.729	1494.733	2.8	1109
GlcN^{Red}(-Ac)-Lac-Ala-Glu-DAP-Ala→DAP(-Ala)-Glu-Ala-Lac-GlcN^{Red}(-Ac)				
GlcN^{Red}(-Ac)-Lac-Ala-Glu-DAP-Ala→DAP(-Ala)-Glu-Ala	1205.576	1205.583	6.2	113
Ala-Glu-DAP-Ala→DAP(-Ala)-Glu-Ala-Lac-GlcN^{Red}(-Ac)				
Glu-DAP-Ala→DAP(-Ala)-Glu-Ala-Lac-GlcN^{Red}(-Ac)	1146.576	1146.580	3.8	295
GlcN^{Red}(-Ac)-Lac-Ala-Glu-DAP-Ala→DAP(-Ala)-Glu				
Glu-DAP-Ala→DAP(-Ala)-Glu-Ala-Lac-GlcN^{Red}(-Ac)	1130.533	1130.539	5.7	219
GlcN^{Red}(-Ac)-Lac-Ala-Glu-DAP-Ala→DAP(-Ala)-Glu				
DAP-Ala→DAP(-Ala)-Glu-Ala-Lac-GlcN^{Red}(-Ac)	1017.530	1017.538	7.3	126
GlcN^{Red}(-Ac)-Lac-Ala-Glu-DAP-Ala→DAP(-Ala)				
DAP-Ala→DAP(-Ala)-Glu-Ala-Lac-GlcN^{Red}(-Ac)	995.475	995.483	7.4	176
GlcN^{Red}(-Ac)-Lac-Ala-Glu-DAP-Ala→DAP(-Ala)				
DAP-Ala→DAP(-Ala)-Glu	647.322	647.329	11.3	149
Glu-DAP-Ala→DAP(-Ala)				
DAP-Glu	302.130	302.135	15.7	91
Glu-DAP				
GlcN(-Ac)	204.089	204.087	-9.0	2307
GlcN(-Ac)				
DAP	182.108	182.110	12.0	134

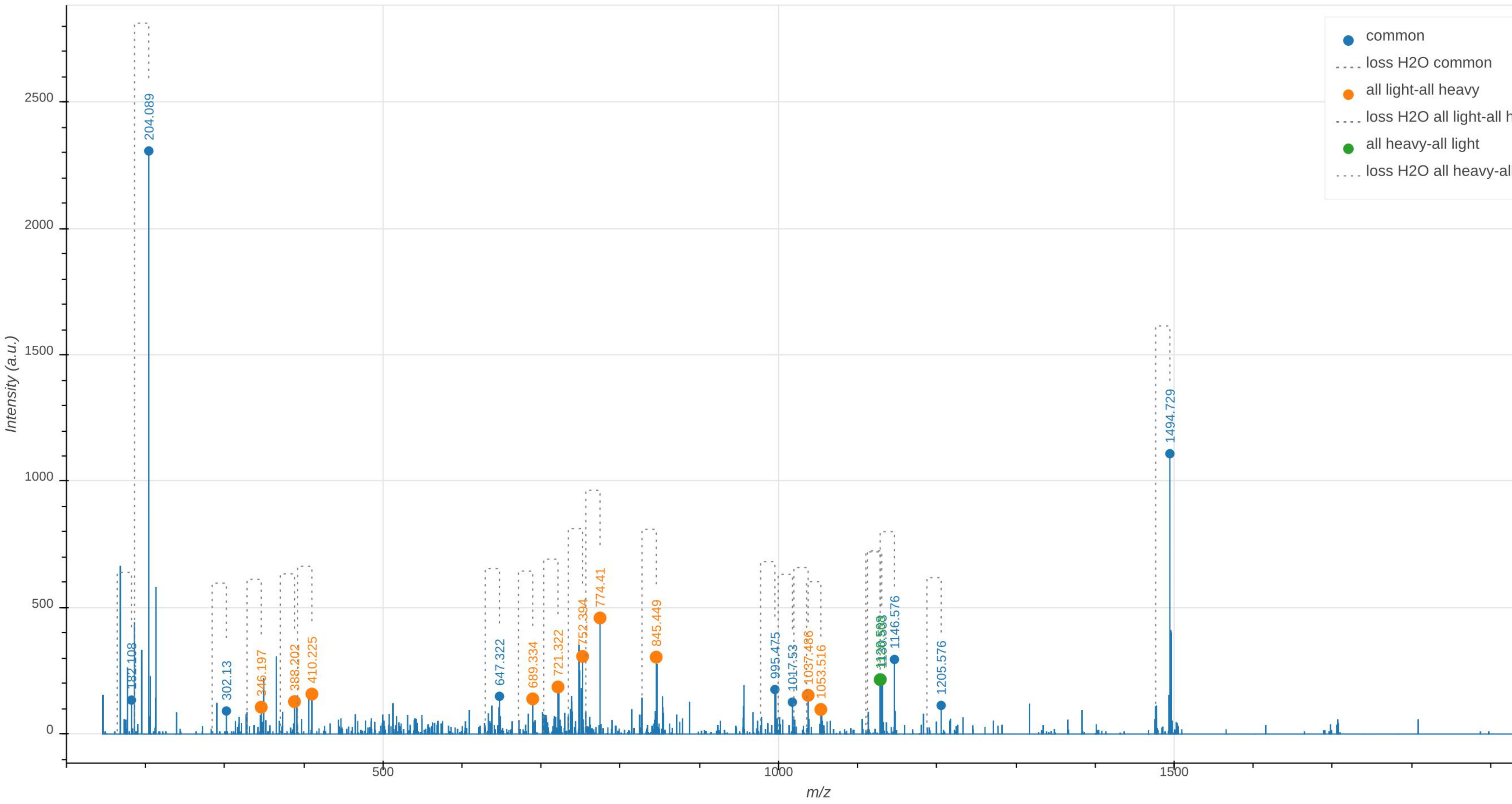

common
loss H2O common
all light-all heavy
loss H2O all light-all h
all heavy-all light
loss H2O all heavy-all
Intensity (a.u.)
m/z
0
500
1000
1500
2000
2500
500
1000
1500
204.089
187.108
302.13
346.197
388.202
410.225
647.322
689.334
721.322
752.394
774.41
845.449
995.475
1017.53
1037.486
1053.516
1146.576
1205.576
1494.729
